# Supplementary material for: Improving prediction of response to neoadjuvant treatment in patients with breast cancer by combining liquid biopsies with multiparametric MRI: protocol of the LIMA study – a multicentre prospective observational cohort study
Source: BMJ Open. 2022 Sep 20;12(9):e061334. doi: 10.1136/bmjopen-2022-061334 (PMC9490628; doi:10.1136/bmjopen-2022-061334)
Supplement: Supplementary data [file bmjopen-2022-061334supp001.pdf]

Supplementary information

**custom UltraSEEK® Breast Cancer Panel[1]**	
**Gene**	**Coverage (missense mutations)**
PIK3CA (33.89% freq in invasive breast cancer[2])	N345K, C420R, E542K, E545K, E545Q, E545A, H1047R, H1047L
TP53 (36.34% freq in invasive breast cancer[2])	R175H, R213, Y220C, R248W, R248W, R248Q, R273C, R273H
AKT1 (4.52% freq in invasive breast cancer[2])	E17K, L52R
ERBB2 (1.68% freq in invasive breast cancer[2])	G309E, G309A, S310F, L755R, L755S, L755_T759del, D769H, D769Y, V777L, V777L, L869R
ESR1 (0.63% freq in invasive breast cancer[2])	A283V, K303R, E380Q, V392I, S436P, V534E, L536R, L536Q, Y537N, Y537S, Y537C, D538G, S576L

**Breast cfDNA Methylation Panel[3]**	
**Gene**	**Genomic location**
AKR1B1	Chr7:134459123
APC	Chr5:112737754
ARHGEF7	Chr13:111115541
BRCA1	Chr17:43125416
COL6A2	Chr21:46098888
GPX7	Chr14:37592244
HIST1H3C	Chr1:52602513
MDGI	Chr17:48578124
RASGRF2	Chr1:13173414
RASSF1A	Chr5:80960894
TM6SF1	Chr3:50340798
FOXA1	Chr5:180591531
SCGB3A1	Chr15:83107646
TMEFF2	Chr2:192194694

1 Giannoudis, A. *et al.* Genomic profiling using the UltraSEEK panel identifies discordancy between paired primary and breast cancer brain metastases and an association with brain metastasis-free survival. *Breast Cancer Res Treat* **190**, 241-253, doi:10.1007/s10549-021-06364-8 (2021).

2 Cerami, E. *et al.* The cBio cancer genomics portal: an open platform for exploring multidimensional cancer genomics data. *Cancer discovery* **2**, 401-404, doi:10.1158/2159-8290.CD-12-0095 (2012).

3 A. Sartori, E. K., D. Irwin, S. Joosse. in *Association for Molecular Pathology 2021* Vol. 23 1567-1649 (Elsevier, 2021).
