# Supplementary material for: Improving prediction of response to neoadjuvant treatment in patients with breast cancer by combining liquid biopsies with multiparametric MRI: protocol of the LIMA study – a multicentre prospective observational cohort study
Source: BMJ Open. 2022 Sep 20;12(9):e061334. doi: 10.1136/bmjopen-2022-061334 (PMC9490628; doi:10.1136/bmjopen-2022-061334)
Supplement: Author's manuscript [file bmjopen-2022-061334.draft_revisions.pdf]

BMJ Open

BMJ Open is committed to open peer review. As part of this commitment we make the peer review history of every article we publish publicly available.

When an article is published we post the peer reviewers' comments and the authors' responses online. We also post the versions of the paper that were used during peer review. These are the versions that the peer review comments apply to.

The versions of the paper that follow are the versions that were submitted during the peer review process. They are not the versions of record or the final published versions. They should not be cited or distributed as the published version of this manuscript.

BMJ Open is an open access journal and the full, final, typeset and author-corrected version of record of the manuscript is available on our site with no access controls, subscription charges or pay-per-view fees (http://bmjopen.bmj.com).

If you have any questions on BMJ Open's open peer review process please email info.bmjopen@bmj.com

BMJ Open

Improving prediction of response to neoadjuvant treatment in patients with breast cancer by combining liquid biopsies with multiparametric MRI: protocol of the LIMA study, a multicenter prospective observational cohort study

Journal:	*BMJ Open*
Manuscript ID	bmjopen-2022-061334
Article Type:	Protocol
Date Submitted by the Author:	26-Jan-2022

Complete List of Authors:	Janssen, Liselore; University Medical Centre Utrecht, Image Sciences Institute<br>Suelmann, Britt; University Medical Centre Utrecht, Department of Medical Oncology<br>Elias, Sjoerd; University Medical Centre Utrecht, Julius Center for Health Sciences and Primary Care<br>Janse, Markus; University Medical Centre Utrecht, Image Sciences Institute<br>van Diest, P.J.; University Medical Centre Utrecht, Department of Pathology<br>van der Wall, Elsken; University Medical Centre Utrecht, Department of Medical Oncology<br>Gilhuijs, Kenneth; University Medical Centre Utrecht, Image Sciences Institute

Keywords:	Breast tumours < ONCOLOGY, CHEMOTHERAPY, Magnetic resonance imaging < RADIOLOGY & IMAGING, Adult oncology < ONCOLOGY, Breast imaging < RADIOLOGY & IMAGING

SCHOLARONE™ Manuscripts

Title: Improving prediction of response to neoadjuvant treatment in patients with breast cancer by combining liquid biopsies with multiparametric MRI: protocol of the LIMA study, a multicenter prospective observational cohort study

Authors: L.M. Janssen[1], B.B.M. Suelmann[2], S.G. Elias[4], M.H.A. Janse[1], P.J. van Diest[5], E. van der Wall[2], K.G.A. Gilhuijs[1].
Corresponding Author: Liselore Janssen
Email corresponding author: janssenliselore@gmail.com

Affiliations
[1]Image Sciences Institute, University Medical Center Utrecht, Utrecht, the Netherlands.
[2]Department of Medical Oncology, University Medical Center Utrecht, Utrecht University, Utrecht, The Netherlands.
[3]Julius Center for Health Sciences and Primary Care, University Medical Center Utrecht, Utrecht, the Netherlands
[4]Department of Pathology, University Medical Centre Utrecht, PO Box 85500, 3508 GA, Utrecht, The Netherlands

Abstract
Introduction The response to neoadjuvant chemotherapy (NAC) in breast cancer has important prognostic implications. Dynamic prediction of tumor regression by NAC may allow for adaption of the treatment plan before completion, or even before the start of treatment. Such predictions may help prevent overtreatment and related toxicity and correct for undertreatment with ineffective regimens. Current imaging methods are not able to fully predict efficacy of NAC. To successfully improve response prediction, tumor biology and heterogeneity as well as treatment-induced changes have to be considered. In the LIMA study, multiparametric magnetic resonance imaging (MRI) will be combined with liquid biopsies. In addition to conventional clinical and pathological information, these methods may give complementary information at multiple time points during treatment.
Aim: To combine multiparametric MRI and liquid biopsies in breast cancer patients to predict Residual Cancer Burden (RCB) after NAC, in adjunct to standard clinico-pathological information. Predictions will be made before the start of NAC, approximately halfway during treatment and after completion of NAC.
Methods In this multicenter prospective observational study we aim to enroll 100 patients. Multiparametric MRI will be performed prior to NAC, approximately halfway and after completion of NAC. Liquid biopsies will be obtained immediately prior to every cycle of chemotherapy and after completion of NAC. The primary endpoint is RCB in the surgical resection specimen following NAC. Collected data will primarily be analyzed using multivariable techniques such as penalized regression techniques.
Ethics and dissemination Medical Research Ethics Committee Utrecht has approved this study (NL67308.041.19). Informed consent will be obtained from each participant. All data is anonymized before publication. The findings of this study will be submitted to international peer-reviewed journals.
Trial registration
The LIMA study is registered on ClinicalTrials.gov under registration number NCT04223492.

For peer review only - http://bmjopen.bmj.com/site/about/guidelines.xhtml

Strengths and limitations of this study

- The LIMA trial aims to improve prediction of response to neoadjuvant treatment in patients with breast cancer by combining liquid biopsies with multiparametric MRI
- LIMA is a prospective multicenter observational trial that includes women with early stage breast cancer in the Netherlands
- The LIMA trial was designed to resemble daily clinical practice which facilitates translation and adds to generalizability of results
- The LIMA trial has a low burden for recruited patients

Keywords

Breast tumors, chemotherapy, magnetic resonance imaging, adult oncology, breast imaging

For peer review only

Introduction

Neoadjuvant chemotherapy (NAC) has become an important treatment strategy for early stage breast cancer patients. Compared to adjuvant chemotherapy, NAC potentially results in less extensive surgery of both breast and axilla, without compromising distant recurrence, breast cancer survival or overall survival (OS) (1-3). The degree of response depends largely on sensitivity to therapy and is known to vary in the different breast cancer subtypes, where the highest pathological complete response (pCR) rate is reached within the human epidermal growth factor receptor 2 (HER2)-positive and the triple negative subtypes (4-7).

With the neoadjuvant approach, the tumor is left *in situ* during chemotherapy, which enables evaluation of treatment efficacy. Whether pCR is achieved has an impact on patient prognosis, although prognostic value may vary depending on pCR definition and tumor subtype (4). However, the binary pCR measure ignores differences in prognosis within patients with residual disease. For a more comprehensive evaluation of tumor response after NAC, the residual cancer burden (RCB) was therefore developed, which has shown to be prognostic in all phenotypic subtypes of breast cancer (8, 9).

Although important for prognosis, evaluation of the response at NAC is typically only provided in the post-NAC surgical resection specimen, leaving only room for tailoring the treatment post-surgery, i.e. adjuvant therapy. In the optimal situation, reliable information on tumor response is obtained during, or even before start of, NAC treatment providing the opportunity to tailor the neoadjuvant and surgical treatment to the observed tumor response.

Different methods for predicting tumor response prior to surgery are available in daily clinical practice, e.g., using dynamic contrast enhanced magnetic resonance imaging (DCE-MRI) of the breast. The sensitivity of CE-MRI for predicting pCR after NAC is reported to range between 65% and 91% and specificity is reported to range from between 81% and 88% (10-13). In clinical practice, these are generally not considered high enough to guide treatment decisions, as missed residual disease and inappropriate adjustment of treatment can have a detrimental effect on patient's prognosis.

A method to improve the accuracy of MRI uses various different imaging protocols in one single session (multiparametric MRI). Hence, the MRI is registers information associated with various aspects of tumor biology (proliferation, angiogenesis and metabolism). By adding diffusion weighted imaging (DWI) to the MRI protocol, intratumoral cellularity can be assessed as well, which may improve the value of MRI before, during and after NAC (14, 15).

However, multiparametric MRI is only able to visualize macroscopic disease. To optimize personalized response monitoring, some provision for analysis of microscopic residual disease is needed as well. Repeat core biopsies of the tumor bed during treatment has, however, proven to be hardly feasible in clinical setting(16).

In contrast, liquid biopsies are minimally invasive and blood samples can contain information from all parts of the tumor, thus potentially capturing intra-tumoral heterogeneity. Liquid biopsies are therefore considered a promising tool for prediction of treatment response. Nonetheless, the technique is not yet part of standard clinical practice during NAC. Blood samples of cancer patients can contain circulating tumor cells (CTCs) and circulating DNA. The total of circulating DNA (cirDNA) can contain DNA from different sources (17). When mutations that are associated with the malignant tumor are found in this cirDNA, this is called circulating tumor (ctDNA). Both the total cirDNA and mutations found in ctDNA can contain important information. In patients with breast cancer who are treated with NAC, the presence

For peer review only - http://bmjopen.bmj.com/site/about/guidelines.xhtml

of CTCs in their blood both prior to NAC and prior to surgery is associated with worse disease-free survival (DFS) and OS (18, 19). In a recent study in triple negative (TN) breast cancer treated with NAC, who had residual disease at surgery, an increasing CTC count after surgery was correlated with inferior distant disease free survival (DDFS), DFS and OS (20).

When serial blood samples are taken during treatment, the short half-life of ctDNA (less than 2 hours) allows for changes to be detected quickly and this facilitates dynamic response prediction (21). Tracking of ctDNA mutations during neoadjuvant treatment can give information on presence and load of residual disease as well as associated risk of distant recurrence and mortality (22). ctDNA analysis during treatment may also detect emerging resistance mechanisms, thus allowing the efficacy of anticancer treatments to be monitored (23, 24). Because driver mutations in breast cancer can be present at very low frequencies, especially in early stages of the disease, highly sensitive assays are necessary. In addition to mutations, epigenetic changes are also important for cancer evolution. Methylation can also be detected in breast cancer patients' blood samples and have additional prognostic value (25), which may add to more accurate prediction of treatment response.

In summary, both MRI and liquid biopsies have been assessed individually confirming their potential to be used in response prediction and evaluation of neoadjuvant breast cancer treatment prior to surgery. Little is known about the combined value of these two techniques to improve prediction of response to NAC so that they can guide personalized treatment decisions. One study by Magbanua et. al.(26) found that adding ctDNA information early during treatment to the MRI predictor functional tumor volume (FTV) resulted in a numerical but not statistically significant increase in performance for pCR prediction. The additive value of ctDNA to MRI to predict response to NAC is thus not unequivocally demonstrated, and further research in this field is required. Our study may add to fine-tuning working hypotheses for follow-up studies that may ultimately lead to practical guidelines, as its design allows for easy translation.

Methods

Study objectives

The primary objective is to explore to what extent the combination of multiparametric MRI, and liquid biopsies prior to, during and after completion of NAC, are able to predict residual cancer burden after NAC in addition to conventional clinical and pathological information.

Secondary objective is to use the strategy from the primary objective to predict alternative outcome measures: ypT0 ypN0 (i.e., absence of invasive cancer and *in situ* cancer in the breast and axillary nodes), ypT0/is ypN0 (i.e., absence of invasive cancer in the breast and axillary nodes, irrespective of ductal carcinoma *in situ*), ypT0/is (i.e., absence of invasive cancer in the breast irrespective of ductal carcinoma *in situ* or nodal involvement) and residual lesion volume on DCE-MRI following NAC.

Study design

This is a prospective multicenter observational study in breast cancer patients undergoing NAC. The study has been approved by the Medical Ethics Review Committee of the University Medical Center Utrecht (19-396, NL67308.041.19). SPIRIT guidelines were followed(27). In the LIMA study, the complementary expertise of investigators in the MRI and liquid biopsy field have been combined into a consortium. The study participants will be recruited in 4 different Dutch hospitals. Potential study participants are screened by their treating physicians. Written informed consent will be obtained from all participants by their physician or research

For peer review only - http://bmjopen.bmj.com/site/about/guidelines.xhtml

nurse. All participants will undergo NAC followed by surgery according to the Dutch oncology guidelines (28). Study duration is from diagnosis of invasive breast cancer until the pathological assessment of the resection specimen after surgery. The LIMA study is registered on ClinicalTrials.gov under registration number NCT04223492.

Patient and public involvement

Patients and public were not involved in study design. Results will not be directly disseminated to participating patients because of the unclear clinical relevance to their individual case. Results will be disseminated according to FAIR principles.

Study population

In order to be eligible to participate for the study, a subject must meet all inclusion criteria and none of the exclusion criteria. We aim to include 100 patients.

Inclusion criteria:

Female patients aged 18 years or older

- Histologically proven invasive breast carcinoma
- Planned to receive NAC (and in case of a HER2-positive tumor: addition of trastuzumab and/or pertuzumab)

Exclusion criteria:

- Breast cancer estrogen receptor (ER)-positive and HER2-negative by immunohistochemistry and Bloom and Richardson grade 1
- Inflammatory breast cancer
- Distant metastases on positron emission computed tomography (PET/CT)
- Prior ipsilateral breast cancer (contralateral breast cancer >5 years ago is allowed)
- Other active malignant disease in the past 5 years (excluded squamous cell or basal cell carcinoma of the skin)
- Pregnancy or lactation
- Contra-indications for MRI according to standard hospital guidelines
- Contra-indications for gadolinium-based contrast-agent, including known prior allergic reaction to any contrast-agent, and renal failure, defined by a glomerular filtration rate < 30 mL/min/1.73m^2

Study procedures

An overview of the study procedures is shown in figure 1. All patients will undergo a PET/CT scan before the start of NAC to ensure no metastases are present at distant sites.

MRI acquisition and analysis

MRI will be performed prior to, during (approximately halfway), and after NAC but before surgery. MRI will take place on 3 Tesla field strength scanners with a standardized scanning protocol. All MRI scans will be centrally revised by an experienced breast radiologist, blinded to predictors and primary outcome. Tumor imaging characteristics including BI-RADS descriptors and tumor dimensions in 3 directions will be recorded in the electronic case report form (eCRF). We will implement robust apparent diffusion coefficient (ADC) mapping using standardization of diffusion weighting factors (b- values). Quantitative imaging features will be extracted automatically from tumor and healthy tissues (reflecting microenvironment). These

For peer review only - http://bmjopen.bmj.com/site/about/guidelines.xhtml

methods will be developed and extended from previous studies (29). Optionally, the impact of adding PET features and MRI conductivity features may be explored.

Liquid biopsies

Blood samples will be taken from the patients before administration of every chemotherapy cycle, and after completion of NAC prior to surgery. Because the optimal time point for liquid biopsy analysis in the neoadjuvant treatment of non-metastatic breast cancer is still unknown, multiple liquid biopsies will be taken at multiple time points over the course of the treatment. This also allows for close monitoring of trends over the course of time.

Blood samples will be drawn into blood collection tubes containing a preservation fluid. The ctDNA blood samples will be centrifuged and stored at -80 °C before further processing. Liquid biopsy analyses take place in the lab of Philips in Eindhoven. All technicians will be blinded to primary and secondary outcome measures, as well as predictors. Every sample has a unique identifier so that technicians are blinded to study participant number and longitudinal order until data collection is completed. For the analysis of the ctDNA a pre-specified mutation- and methylation panel will be used. We will predominantly rely on a mass spectroscopy system. Since mass spectroscopy is not suited to detect copy number variations, we will use digital droplet PCR (ddPCR) for this purpose. The ddPCR method can also be used to detect mutations that are not being picked up by the mass spectroscopy system. To isolate and analyze CTCs, the blood will be filtered to reduce the amount of candidate cells by a size and compressibility filter step. After staining, the cells are scanned on a slide to identify the cells which meet the criteria to classify as CTC.

Pathological evaluation

All pathology review will be centralized at UMC Utrecht and performed by a dedicated breast pathologist with >20 years of experience. Central review will be performed on the pre-NAC needle biopsies and the post-NAC surgical resection specimen. Blinding to results for research purposes will be performed, i.e., the researchers that assess the outcome variables (pathology) do not have access to the potential candidate predictors and the other way around.

Diagnostic biopsy Tumor sections will be stained by hematoxylin and eosin (H&E) staining for initial pathology diagnosis including histologic type and grade according to the Nottingham modification of the Bloom and Richardson method. Immunohistochemistry staining for tumor markers will be routinely performed on the most representative paraffin block. ER, PR and HER2 will be interpreted according to Dutch guidelines (28). ER and PRs receptor are considered positive if >10% of nuclei stain positive. Tumors with 3+ HER2 score (strong homogeneous membrane staining in >10% of tumor cells) or HER2 gene amplification are considered HER2 positive on central revision.

Surgical resection specimen Management of the resection specimens will be carried out according to the routine clinical protocol. RCB takes the dimensions of the primary tumor bed into account, as well as cellularity, percentage of in situ disease, number of positive lymph nodes and diameter of the largest lymph node metastases. These items will be reviewed in the surgical resection specimen by a trained pathologist. Calculation of the Residual Cancer Burden will be done according to the guidelines and using the calculator provided by the MD Anderson website(30).

For peer review only - http://bmjopen.bmj.com/site/about/guidelines.xhtml

Data collection & safety reporting

Treatment regimen and patient characteristics including age, height, weight, menopausal status and AJCC TNM stage will be recorded in the eCRF. For the eCRF a Good Clinical Practice (GCP)-compliant data capture tool will be used, which has direct input validation, edit checks and automatic saving. Personal data will be saved in an encrypted software system with two-factor authentication and limited access for designated study team members only. This study will follow the FAIR principles in handling and storage of data (31). A data safety monitoring board is not implemented because the study is in the negligible-risk category. For this reason, only two adverse events that can be related to the study procedures will be reported as (serious) adverse events: allergic reactions to contrast agents that are administered during the MRI scans and (thrombo)phlebitis as a result of the intravenous catheter. According to regulations, a medical doctor is always present at the MRI unit when contrast is given. Study monitoring is coordinated by the sponsor and bi-annual monitoring visits are planned.

Statistical analysis plan

A formal sample size and power calculation is impossible for this type of study with a large number of candidate predictor features in relation to the number patients, because meaningful (co-)variance data is lacking to feed informative simulation studies. Nevertheless, similar studies of this size have succeeded in generating clinically meaningful predictive signatures (32). Furthermore, our primary endpoint (RCB) is continuous, increasing the effective sample size compared to a binary outcome (such as pCR).

The primary analysis population will include all patients who receive at least one cycle of neo-adjuvant treatment and have the primary outcome assessed (i.e., residual breast cancer burden). Patterns of missing data will be inspected and if necessary we will use established methods for multiple imputation to account for missing data under the missing at random (MAR) assumption.

To meet our primary objective we will estimate the over-optimism corrected mean square error and associated 95% confidence intervals for predicting RCB in the primary analysis population using all candidate predictors from the clinical data, biopsy data and imaging data with or without the features from the liquid biopsies. These scenarios are tested at three time points: before, half way through and at the end of NAC treatment. We will use the prediction scenarios with and without liquid biopsies features to examine their additive value to the MRI-clinical-pathology-based model.

To develop the optimal and most parsimonious prediction model for each scenario, we will primarily make use of Least Absolute Shrinkage and Selection Operator (LASSO) penalized linear regression techniques, using bootstrapping to obtain the penalty value that minimizes the mean square error in RCB prediction. This will be repeated in each multiple imputation dataset, and the optimal models from each imputation dataset will then be averaged to obtain one final optimal model for each analyzed scenario. We will repeat all these modelling steps under an additional bootstrap resampling scheme for an additional internal validation step to optimally correct for over-optimism.

Secondary to the estimation of the mean square error of the models, we will assess the models' performance in other ways as well, including: 1) agreement between predicted and actual observed RCB to assess calibration using scatterplots and linear regression analysis; 2) performance of the prediction models when the predictions of RCB as a continuous measure

For peer review only - http://bmjopen.bmj.com/site/about/guidelines.xhtml

are compared to clinically relevant subgroups of actual RCB using receiver operating curve (ROC) curves (discrimination) and decision curve analysis (net benefit). For our secondary objectives we will use similar data-analysis approaches.

Discussion

With the neoadjuvant approach, the tumor is left *in situ* during chemotherapy. The extent to which the tumor of an individual patients responds to NAC is highly variable. This variability in response means a certain NAC regimen could be overtreatment in one patient, but undertreatment in another. To define the right treatment approach for an individual patients, and to correctly balance the treatment related side-effects and oncological safety, accurate prediction of response is essential. Response prediction could be used to personalize treatment for breast cancer treated with NAC in different scenarios. After completion of NAC, but before surgery, reliable tumor response evaluation is essential for facilitating de-escalation of the surgical treatment of both breast and axilla. If this evaluation is accurate enough, a wait-and-see approach may even be imaginable, sparing patients surgery-associated morbidity.

When response to NAC is assessed at earlier time points during treatment, it can provide a different set of opportunities for tailoring the treatment to individual patients' needs. An inadequate tumor response at interim evaluation may guide the treating physicians to opt for a different (non-cross resistant) chemotherapy regimen, choose a different type of systemic treatment, or adapt (the timing of) surgical intervention. Chemotherapy treatment is associated with comorbidities and reduced quality of life in breast cancer patients. Excellent response at interim evaluation could also be a reason for adapting (the timing of) surgical intervention or may make chemotherapy de-escalation possible, thereby sparing patients unnecessary side-effects.

Especially prediction of tumor response before start of any treatment is challenging, but could have a major impact on determining the treatment strategy. Leaving the tumor *in situ* during NAC can carry risks in aggressive tumors that will not respond to NAC. If this (lack of) response to NAC could be reliably predicted beforehand, more effective treatment options may be adopted.

At this point, however, no method for response prediction available in clinical practice is deemed accurate enough to guide this personalized treatment approach. New strategies for predicting response to NAC include image guided tumor bed biopsy for detecting pCR in the breast after NAC in patients with partial or complete radiologic response. Unfortunately, studies have shown relatively high false negative rates ranging from 17.8-37% for detecting pCR (defined as ypT0), which means tumor bed biopsies cannot (yet) be used to safely omit surgery after NAC. This may be explained by the fact that tissue biopsies are prone to sampling error, due to intra-tumoral spatial heterogeneity (33). The invasive nature of tissue biopsies is also a drawback for clinical implementation.

Both multiparametric MRI and liquid biopsies are non-invasive methods for the evaluation of response that are valuable for the prediction of response to NAC. In the LIMA study these techniques are uniquely combined to fully exploit the complementary information they hold.

A study by Magbanua et al. (26) studied the combined use of ctDNA and MRI to predict pCR in patients included in the I-SPY 2 TRIAL (NCT01042379). They found an increase in area under the curve (AUC) by adding ctDNA to an MRI-derived functional tumor volume model after 3 weeks of paclitaxel-based therapy, but the increase did not reach statistical significance.

For peer review only - http://bmjopen.bmj.com/site/about/guidelines.xhtml

Functional tumor volume and ctDNA both did remain significant predictors of distant recurrence free survival in an exploratory multivariable analysis. Our study may add to these results on several aspects. We opted for a study design that is as close to clinical practice as possible and does not include regular study visits since blood is drawn from the intravenous catheter that is already in place during regular chemotherapy treatment appointments. Our patients are treated according to the most recent standard clinical guidelines. Therefore our study design reflects daily clinical practice, which will add to the generalizability of our findings.

Secondly, the trend that values of liquid biopsy predictors follow between different timepoints may hold important information, apart from these values themselves. Because our study has a liquid biopsy data point at every chemotherapy cycle, meaningful trends can be obtained which could lead to better predictions. Thus, we also account for the fact that the optimal time points and intervals to assess ctDNA in the neoadjuvant setting are currently unknown.

There are a few useful things to consider in translating this study design to a clinical practice situation. Blood samples are analyzed in an external lab which may come with some logistical challenges. Additionally, specific patients are excluded: patients with B&R grade 1 hormone receptor positive breast cancer are excluded because of the is the poor NAC treatment results that have been reached for this subtype, and the proposed systemic treatment de-escalation prescribed in current guidelines. Patients with inflammatory breast cancer and recent other malignancies are excluded because these could lead to misinterpretation of ctDNA results. Pregnant or lactating women are excluded because their breast tissue on MRI would be influenced too much. Patients with a contra-indication for MRI or contrast are excluded for their safety.

This study is one of the first to combine multiparametric MRI with liquid biopsies to predict response to neoadjuvant chemotherapy in breast cancer. If the results of this study show proof-of-concept for combining these two techniques for accurate response prediction, larger follow-up studies can be designed to validate the value of these combined modalities in daily clinical practice.

For peer review only

Ethics and dissemination

Medical Research Ethics Committee Utrecht has approved this study (NL67308.041.19). Informed consent will be obtained from each participant. All data is anonymized before publication. The findings of this study will be submitted to international peer-reviewed journals.

Word count: 3566

Abbreviations

ADC: apparent diffusion coefficient; AUC: area under the curve; cirDNA: circulating DNA; CNN: convolutional neural networks; CTC: circulating tumor cell; ctDNA: circulating tumor DNA; DCE: dynamic contrast enhanced; ddPCR: digital droplet polymerase chain reaction; DFS: disease-free survival; DDFS: distant disease free survival; DWI: diffusion weighted imaging; eCRF: electronic case report form; ER: estrogen receptor; FFPE: formalin-fixed paraffin-embedded; FTV: functional tumor volume; GCP: good clinical practice; HER2: human epidermal growth factor receptor 2; H&E: hematoxylin and eosin; LASSO: Least Absolute Shrinkage and Selection Operator; MAR: missing at random; MRI: magnetic resonance imaging; NAC: neoadjuvant chemotherapy; OS: overall survival; pCR: pathological complete response; PET/CT: positron emission computed tomography; PR: progesterone receptor; RCB: residual cancer burden; RFS: recurrence-free survival; ROC: receiver operating curve

Competing interests

The authors declare that they have no competing interests.

Funding

Funding from the European Union Horizon 2020 research and innovation program under grant agreement no. 755333 (LIMA). Funder was not involved in study design.

Authors' contributions

Guarantors of integrity of entire study, L.M.J., K.G.A.G.; study concepts/study design or data acquisition: all authors; future data analysis/interpretation, all authors; future statistical analysis: L.M.J., S.E., K.G.A.G.; manuscript drafting or manuscript revision for important intellectual content, all authors; approval of final version of submitted manuscript, all authors; literature research, L.M.J., P.V.D., E.V.D.W. ; clinical studies, L.M.J., K.G.A.G., P.V.D., E.V.D.W.

Acknowledgements

We would like to acknowledge dr. W.B. Veldhuis for input from a radiological standpoint and we are grateful for the support of the trial bureau of division Imaging&Oncology of the UMC Utrecht, with a special thanks to Shanta Kalaykhan.

This study is funded by European Union Horizon 2020 research and innovation program under grant agreement no. 755333 (LIMA). Other members of this project are employees of Institut national de la santé et de la recherche médicale (INSERM), Montpellier Cancer Institute France, Angle Europe Limited, ALS Automated Lab Solutions GmbH, AGENA Bioscience GmbH, DiaDX, Stilla Technologies, Philips Electronics Nederland B.V. and Philips GmbH.

For peer review only - http://bmjopen.bmj.com/site/about/guidelines.xhtml

Literature

1. Asselain B, Barlow W, Bartlett J, Bergh J, Bergsten-Nordström E, Bliss J, et al. Long-term outcomes for neoadjuvant versus adjuvant chemotherapy in early breast cancer: meta-analysis of individual patient data from ten randomised trials. The Lancet Oncology. 2018;19(1):27-39.
2. El Hage Chehade H, Headon H, El Tokhy O, Heeney J, Kasem A, Mokbel K. Is sentinel lymph node biopsy a viable alternative to complete axillary dissection following neoadjuvant chemotherapy in women with node-positive breast cancer at diagnosis? An updated meta-analysis involving 3,398 patients. The American Journal of Surgery. 2016;212(5):969-81.
3. Simons JM, Maaskant-Braat AJG, Luiten EJT, Leidenius MHK, van Nijnatten TJA, Boelens PG, et al. Patterns of axillary staging and management in clinically node positive breast cancer patients treated with neoadjuvant systemic therapy: Results of a survey amongst breast cancer specialists. European Journal of Surgical Oncology. 2020;46(1):53-8.
4. Cortazar P, Zhang L, Untch M, Mehta K, Costantino JP, Wolmark N, et al. Pathological complete response and long-term clinical benefit in breast cancer: the CTNeoBC pooled analysis. Lancet (London, England). 2014;384(9938):164-72.
5. Haque W, Verma V, Hatch S, Suzanne Klimberg V, Brian Butler E, Teh BS. Response rates and pathologic complete response by breast cancer molecular subtype following neoadjuvant chemotherapy. Breast Cancer Res Treat. 2018;170(3):559-67.
6. von Minckwitz G, Untch M, Blohmer JU, Costa SD, Eidtmann H, Fasching PA, et al. Definition and impact of pathologic complete response on prognosis after neoadjuvant chemotherapy in various intrinsic breast cancer subtypes. J Clin Oncol. 2012;30(15):1796-804.
7. Zeidman M, Alberty-Oller JJ, Ru M, Pisapati KV, Moshier E, Ahn S, et al. Use of neoadjuvant versus adjuvant chemotherapy for hormone receptor-positive breast cancer: a National Cancer Database (NCDB) study. Breast Cancer Res Treat. 2020.
8. Symmans WF, Wei C, Gould R, Yu X, Zhang Y, Liu M, et al. Long-Term Prognostic Risk After Neoadjuvant Chemotherapy Associated With Residual Cancer Burden and Breast Cancer Subtype. J Clin Oncol. 2017;35(10):1049-60.
9. Yau C, Van Der Noordaa M, Wei J, Osdoit M, Reyal F, Hamy AS, et al. Residual cancer burden after neoadjuvant therapy and long-term survival outcomes in breast cancer: A multi-center pooled analysis. Cancer Research. 2020;80(4).
10. Liu Q, Wang C, Li P, Liu J, Huang G, Song S. The Role of (18)F-FDG PET/CT and MRI in Assessing Pathological Complete Response to Neoadjuvant Chemotherapy in Patients with Breast Cancer: A Systematic Review and Meta-Analysis. BioMed research international. 2016;2016:3746232.
11. Tang S, Xiang C, Yang Q. The diagnostic performance of CESM and CE-MRI in evaluating the pathological response to neoadjuvant therapy in breast cancer: a systematic review and meta-analysis. The British journal of radiology. 2020;93(1112):20200301.
12. Cheng Q, Huang J, Liang J, Ma M, Ye K, Shi C, et al. The Diagnostic Performance of DCE-MRI in Evaluating the Pathological Response to Neoadjuvant Chemotherapy in Breast Cancer: A Meta-Analysis. Front Oncol. 2020;10:93.
13. Virostko J, Hainline A, Kang H, Arlinghaus LR, Abramson RG, Barnes SL, et al. Dynamic contrast-enhanced magnetic resonance imaging and diffusion-weighted magnetic resonance imaging for predicting the response of locally advanced breast cancer to neoadjuvant therapy: a meta-analysis. Journal of medical imaging (Bellingham, Wash). 2017;5(1):011011.
14. Li W, Newitt DC, Wilmes LJ, Jones EF, Arasu V, Gibbs J, et al. Additive value of diffusion-weighted MRI in the I-SPY 2 TRIAL. J Magn Reson Imaging. 2019.
15. Gu YL, Pan SM, Ren J, Yang ZX, Jiang GQ. Role of Magnetic Resonance Imaging in Detection of Pathologic Complete Remission in Breast Cancer Patients Treated With Neoadjuvant Chemotherapy: A Meta-analysis. Clinical breast cancer. 2017;17(4):245-55.
16. van Loevezijn AA, van der Noordaa MEM, van Werkhoven ED, Loo CE, Winter-Warnars GAO, Wiersma T, et al. Minimally Invasive Complete Response Assessment of the Breast After Neoadjuvant Systemic Therapy for Early Breast Cancer (MICRA trial): Interim Analysis of a Multicenter Observational Cohort Study. Ann Surg Oncol. 2021;28(6):3243-53.

For peer review only - http://bmjopen.bmj.com/site/about/guidelines.xhtml

17. Thierry AR, El Messaoudi S, Gahan PB, Anker P, Stroun M. Origins, structures, and functions of circulating DNA in oncology. Cancer Metastasis Rev. 2016;35(3):347-76.
18. Bidard FC, Michiels S, Riethdorf S, Mueller V, Esserman LJ, Lucci A, et al. Circulating Tumor Cells in Breast Cancer Patients Treated by Neoadjuvant Chemotherapy: A Meta-analysis. Journal of the National Cancer Institute. 2018;110(6):560-7.
19. Zhang L, Riethdorf S, Wu G, Wang T, Yang K, Peng G, et al. Meta-analysis of the prognostic value of circulating tumor cells in breast cancer. Clin Cancer Res. 2012;18(20):5701-10.
20. Radovich M, Jiang G, Hancock BA, Chitambar C, Nanda R, Falkson C, et al. Association of Circulating Tumor DNA and Circulating Tumor Cells After Neoadjuvant Chemotherapy With Disease Recurrence in Patients With Triple-Negative Breast Cancer: Preplanned Secondary Analysis of the BRE12-158 Randomized Clinical Trial. JAMA Oncology. 2020.
21. Diehl F, Schmidt K, Choti MA, Romans K, Goodman S, Li M, et al. Circulating mutant DNA to assess tumor dynamics. Nature Medicine. 2008;14(9):985-90.
22. Riva F, Bidard FC, Houy A, Saliou A, Madic J, Rampanou A, et al. Patient-Specific Circulating Tumor DNA Detection during Neoadjuvant Chemotherapy in Triple-Negative Breast Cancer. Clin Chem. 2017;63(3):691-9.
23. Ma F, Zhu W, Guan Y, Yang L, Xia X, Chen S, et al. ctDNA dynamics: a novel indicator to track resistance in metastatic breast cancer treated with anti-HER2 therapy. Oncotarget. 2016;7(40):66020-31.
24. Hu ZY, Xie N, Tian C, Yang X, Liu L, Li J, et al. Identifying Circulating Tumor DNA Mutation Profiles in Metastatic Breast Cancer Patients with Multiline Resistance. EBioMedicine. 2018;32:111-8.
25. Panagopoulou M, Karaglani M, Balgkouranidou I, Biziota E, Koukaki T, Karamitrousis E, et al. Circulating cell-free DNA in breast cancer: size profiling, levels, and methylation patterns lead to prognostic and predictive classifiers. Oncogene. 2019.
26. Magbanua MJM, Li W, Wolf DM, Yau C, Hirst GL, Swigart LB, et al. Circulating tumor DNA and magnetic resonance imaging to predict neoadjuvant chemotherapy response and recurrence risk. NPJ breast cancer. 2021;7(1):32.
27. Chan A-W, Tetzlaff JM, Gøtzsche PC, Altman DG, Mann H, Berlin JA, et al. SPIRIT 2013 explanation and elaboration: guidance for protocols of clinical trials. BMJ : British Medical Journal. 2013;346:e7586.
28. Nederland Ik. Landelijke richtlijn Borstkanker 2017 [Available from: www.oncoline.nl.
29. Aerts HJ, Velazquez ER, Leijenaar RT, Parmar C, Grossmann P, Carvalho S, et al. Decoding tumour phenotype by noninvasive imaging using a quantitative radiomics approach. Nat Commun. 2014;5:4006.
30. Center MAC. Residual Cancer Burden Calculator [cited 2018. Available from: http://www3.mdanderson.org/app/medcalc/index.cfm?pagename=jsconvert3.
31. Wilkinson MD, Dumontier M, Aalbersberg IJ, Appleton G, Axton M, Baak A, et al. The FAIR Guiding Principles for scientific data management and stewardship. Scientific Data. 2016;3(1):160018.
32. van 't Veer LJ, Dai H, van de Vijver MJ, He YD, Hart AA, Mao M, et al. Gene expression profiling predicts clinical outcome of breast cancer. Nature. 2002;415(6871):530-6.
33. Yates LR, Gerstung M, Knappskog S, Desmedt C, Gundem G, Van Loo P, et al. Subclonal diversification of primary breast cancer revealed by multiregion sequencing. Nat Med. 2015;21(7):751-9.

For peer review only - http://bmjopen.bmj.com/site/about/guidelines.xhtml

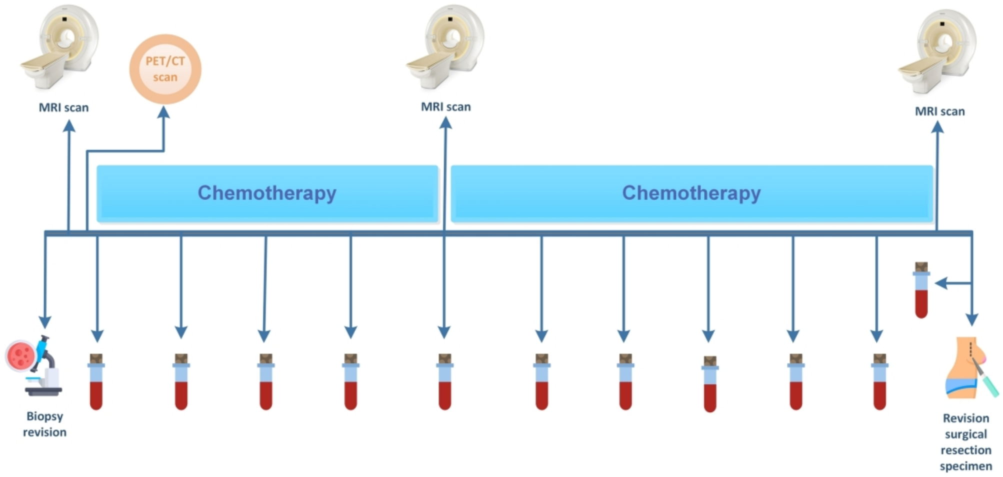

Figure 1: Study procedures

212x102mm (300 x 300 DPI)

STANDARD PROTOCOL ITEMS: RECOMMENDATIONS FOR INTERVENTIONAL TRIALS

SPIRIT 2013 Checklist: Recommended items to address in a clinical trial protocol and related documents*

Section/item	ItemNo	Description	Addresed on page number
**Administrative information**			
Title	1	Descriptive title identifying the study design, population, interventions, and, if applicable, trial acronym	1
Trial registration	2a	Trial identifier and registry name. If not yet registered, name of intended registry	1,5
	2b	All items from the World Health Organization Trial Registration Data Set	NA
Protocol version	3	Date and version identifier	NA
Funding	4	Sources and types of financial, material, and other support	11
Roles and responsibilities	5a	Names, affiliations, and roles of protocol contributors	11
	5b	Name and contact information for the trial sponsor	1
	5c	Role of study sponsor and funders, if any, in study design; collection, management, analysis, and interpretation of data; writing of the report; and the decision to submit the report for publication, including whether they will have ultimate authority over any of these activities	11
	5d	Composition, roles, and responsibilities of the coordinating centre, steering committee, endpoint adjudication committee, data management team, and other individuals or groups overseeing the trial, if applicable (see Item 21a for data monitoring committee)	11

For peer review only

**Introduction**			
Background and rationale	6a	Description of research question and justification for undertaking the trial, including summary of relevant studies (published and unpublished) examining benefits and harms for each intervention	3-4
	6b	Explanation for choice of comparators	3-4
Objectives	7	Specific objectives or hypotheses	4
Trial design	8	Description of trial design including type of trial (eg, parallel group, crossover, factorial, single group), allocation ratio, and framework (eg, superiority, equivalence, noninferiority, exploratory)	4-5
**Methods: Participants, interventions, and outcomes**			
Study setting	9	Description of study settings (eg, community clinic, academic hospital) and list of countries where data will be collected. Reference to where list of study sites can be obtained	4-5
Eligibility criteria	10	Inclusion and exclusion criteria for participants. If applicable, eligibility criteria for study centres and individuals who will perform the interventions (eg, surgeons, psychotherapists)	5
Interventions	11a	Interventions for each group with sufficient detail to allow replication, including how and when they will be administered	5-7
	11b	Criteria for discontinuing or modifying allocated interventions for a given trial participant (eg, drug dose change in response to harms, participant request, or improving/worsening disease)	NA
	11c	Strategies to improve adherence to intervention protocols, and any procedures for monitoring adherence (eg, drug tablet return, laboratory tests)	NA
	11d	Relevant concomitant care and interventions that are permitted or prohibited during the trial	NA

For peer review only - http://bmjopen.bmj.com/site/about/guidelines.xhtml

Outcomes	12	Primary, secondary, and other outcomes, including the specific measurement variable (eg, systolic blood pressure), analysis metric (eg, change from baseline, final value, time to event), method of aggregation (eg, median, proportion), and time point for each outcome. Explanation of the clinical relevance of chosen efficacy and harm outcomes is strongly recommended	4
Participant timeline	13	Time schedule of enrolment, interventions (including any run-ins and washouts), assessments, and visits for participants. A schematic diagram is highly recommended (see Figure)	6
Sample size	14	Estimated number of participants needed to achieve study objectives and how it was determined, including clinical and statistical assumptions supporting any sample size calculations	5
Recruitment	15	Strategies for achieving adequate participant enrolment to reach target sample size	4-5
**Methods: Assignment of interventions (for controlled trials)**			
Allocation:			
Sequence generation	16a	Method of generating the allocation sequence (eg, computer-generated random numbers), and list of any factors for stratification. To reduce predictability of a random sequence, details of any planned restriction (eg, blocking) should be provided in a separate document that is unavailable to those who enrol participants or assign interventions	NA
Allocation concealment mechanism	16b	Mechanism of implementing the allocation sequence (eg, central telephone; sequentially numbered, opaque, sealed envelopes), describing any steps to conceal the sequence until interventions are assigned	NA
Implementation	16c	Who will generate the allocation sequence, who will enrol participants, and who will assign participants to interventions	NA
Blinding (masking)	17a	Who will be blinded after assignment to interventions (eg, trial participants, care providers, outcome assessors, data analysts), and how	6-7

	17b	If blinded, circumstances under which unblinding is permissible, and procedure for revealing a participant's allocated intervention during the trial	NA
**Methods: Data collection, management, and analysis**			
Data collection methods	18a	Plans for assessment and collection of outcome, baseline, and other trial data, including any related processes to promote data quality (eg, duplicate measurements, training of assessors) and a description of study instruments (eg, questionnaires, laboratory tests) along with their reliability and validity, if known. Reference to where data collection forms can be found, if not in the protocol	7
	18b	Plans to promote participant retention and complete follow-up, including list of any outcome data to be collected for participants who discontinue or deviate from intervention protocols	NA
Data management	19	Plans for data entry, coding, security, and storage, including any related processes to promote data quality (eg, double data entry; range checks for data values). Reference to where details of data management procedures can be found, if not in the protocol	7
Statistical methods	20a	Statistical methods for analysing primary and secondary outcomes. Reference to where other details of the statistical analysis plan can be found, if not in the protocol	7-8
	20b	Methods for any additional analyses (eg, subgroup and adjusted analyses)	7-8
	20c	Definition of analysis population relating to protocol non-adherence (eg, as randomised analysis), and any statistical methods to handle missing data (eg, multiple imputation)	7-8
**Methods: Monitoring**			
Data monitoring	21a	Composition of data monitoring committee (DMC); summary of its role and reporting structure; statement of whether it is independent from the sponsor and competing interests; and reference to where further details about its charter can be found, if not in the protocol. Alternatively, an explanation of why a DMC is not needed	7

	21b	Description of any interim analyses and stopping guidelines, including who will have access to these interim results and make the final decision to terminate the trial	NA
Harms	22	Plans for collecting, assessing, reporting, and managing solicited and spontaneously reported adverse events and other unintended effects of trial interventions or trial conduct	7
Auditing	23	Frequency and procedures for auditing trial conduct, if any, and whether the process will be independent from investigators and the sponsor	7
**Ethics and dissemination**			
Research ethics approval	24	Plans for seeking research ethics committee/institutional review board (REC/IRB) approval	4
Protocol amendments	25	Plans for communicating important protocol modifications (eg, changes to eligibility criteria, outcomes, analyses) to relevant parties (eg, investigators, REC/IRBs, trial participants, trial registries, journals, regulators)	4
Consent or assent	26a	Who will obtain informed consent or assent from potential trial participants or authorised surrogates, and how (see Item 32)	5
	26b	Additional consent provisions for collection and use of participant data and biological specimens in ancillary studies, if applicable	5
Confidentiality	27	How personal information about potential and enrolled participants will be collected, shared, and maintained in order to protect confidentiality before, during, and after the trial	7,11
Declaration of interests	28	Financial and other competing interests for principal investigators for the overall trial and each study site	11
Access to data	29	Statement of who will have access to the final trial dataset, and disclosure of contractual agreements that limit such access for investigators	NA

For peer review only

Ancillary and post-trial care	30	Provisions, if any, for ancillary and post-trial care, and for compensation to those who suffer harm from trial participation	NA
Dissemination policy	31a	Plans for investigators and sponsor to communicate trial results to participants, healthcare professionals, the public, and other relevant groups (eg, via publication, reporting in results databases, or other data sharing arrangements), including any publication restrictions	11
	31b	Authorship eligibility guidelines and any intended use of professional writers	NA
	31c	Plans, if any, for granting public access to the full protocol, participant-level dataset, and statistical code	NA
**Appendices**			
Informed consent materials	32	Model consent form and other related documentation given to participants and authorised surrogates	Additional files
Biological specimens	33	Plans for collection, laboratory evaluation, and storage of biological specimens for genetic or molecular analysis in the current trial and for future use in ancillary studies, if applicable	NA

*It is strongly recommended that this checklist be read in conjunction with the SPIRIT 2013 Explanation & Elaboration for important clarification on the items. Amendments to the protocol should be tracked and dated. The SPIRIT checklist is copyrighted by the SPIRIT Group under the Creative Commons "Attribution-NonCommercial-NoDerivs 3.0 Unported" license.

For peer review only

Improving prediction of response to neoadjuvant treatment in patients with breast cancer by combining liquid biopsies with multiparametric MRI: protocol of the LIMA study, a multicenter prospective observational cohort study

Journal:	*BMJ Open*
Manuscript ID	bmjopen-2022-061334.R1
Article Type:	Protocol
Date Submitted by the Author:	21-Jul-2022
Complete List of Authors:	Janssen, Liselore; University Medical Centre Utrecht, Image Sciences Institute<br>Suelmann, Britt; University Medical Centre Utrecht, Department of Medical Oncology<br>Elias, Sjoerd; University Medical Centre Utrecht, Julius Center for Health Sciences and Primary Care<br>Janse, Markus; University Medical Centre Utrecht, Image Sciences Institute<br>van Diest, P.J.; University Medical Centre Utrecht, Department of Pathology<br>van der Wall, Elsken; University Medical Centre Utrecht, Department of Medical Oncology<br>Gilhuijs, Kenneth; University Medical Centre Utrecht, Image Sciences Institute
**Primary Subject Heading**:	Oncology
Secondary Subject Heading:	Radiology and imaging
Keywords:	Breast tumours < ONCOLOGY, CHEMOTHERAPY, Magnetic resonance imaging < RADIOLOGY & IMAGING, Adult oncology < ONCOLOGY, Breast imaging < RADIOLOGY & IMAGING

Title: Improving prediction of response to neoadjuvant treatment in patients with breast cancer by combining liquid biopsies with multiparametric MRI: protocol of the LIMA study, a multicenter prospective observational cohort study

Authors: L.M. Janssen[1], B.B.M. Suelmann[2], S.G. Elias[4], M.H.A. Janse[1], P.J. van Diest[5], E. van der Wall[2], K.G.A. Gilhuijs[1].
Corresponding Author: Kenneth Gilhuijs
Email corresponding author: K.G.A.Gilhuijs@umcutrecht.nl

Affiliations
[1]Image Sciences Institute, University Medical Center Utrecht, Utrecht, the Netherlands.
[2]Department of Medical Oncology, University Medical Center Utrecht, Utrecht University, Utrecht, The Netherlands.
[3]Julius Center for Health Sciences and Primary Care, University Medical Center Utrecht, Utrecht, the Netherlands
[4]Department of Pathology, University Medical Centre Utrecht, PO Box 85500, 3508 GA, Utrecht, The Netherlands

For peer review only

Abstract
Introduction The response to neoadjuvant chemotherapy (NAC) in breast cancer has important prognostic implications. Dynamic prediction of tumor regression by NAC may allow for adaption of the treatment plan before completion, or even before the start of treatment. Such predictions may help prevent overtreatment and related toxicity and correct for undertreatment with ineffective regimens. Current imaging methods are not able to fully predict efficacy of NAC. To successfully improve response prediction, tumor biology and heterogeneity as well as treatment-induced changes have to be considered. In the LIMA study, multiparametric magnetic resonance imaging (MRI) will be combined with liquid biopsies. In addition to conventional clinical and pathological information, these methods may give complementary information at multiple time points during treatment.
Aim: To combine multiparametric MRI and liquid biopsies in breast cancer patients to predict Residual Cancer Burden (RCB) after NAC, in adjunct to standard clinico-pathological information. Predictions will be made before the start of NAC, approximately halfway during treatment and after completion of NAC.
Methods In this multicenter prospective observational study we aim to enroll 100 patients. Multiparametric MRI will be performed prior to NAC, approximately halfway and after completion of NAC. Liquid biopsies will be obtained immediately prior to every cycle of chemotherapy and after completion of NAC. The primary endpoint is RCB in the surgical resection specimen following NAC. Collected data will primarily be analyzed using multivariable techniques such as penalized regression techniques.
Ethics and dissemination Medical Research Ethics Committee Utrecht has approved this study (NL67308.041.19). Informed consent will be obtained from each participant. All data is anonymized before publication. The findings of this study will be submitted to international peer-reviewed journals.
Trial registration
The LIMA study is registered on ClinicalTrials.gov under registration number NCT04223492.

For peer review only - http://bmjopen.bmj.com/site/about/guidelines.xhtml

Strengths and limitations of this study

- The LIMA trial aims to improve prediction of response to neoadjuvant treatment in patients with breast cancer by combining liquid biopsies with multiparametric MRI
- LIMA is a prospective multicenter observational trial that includes women with early stage breast cancer in the Netherlands
- The LIMA trial was designed to resemble daily clinical practice which facilitates translation and adds to generalizability of results
- The LIMA trial has a low burden for recruited patients

Keywords
Breast tumors, chemotherapy, magnetic resonance imaging, adult oncology, breast imaging

For peer review only

Introduction

Neoadjuvant chemotherapy (NAC) has become an important treatment strategy for early stage breast cancer patients. Compared to adjuvant chemotherapy, NAC potentially results in less extensive surgery of both breast and axilla, without compromising distant recurrence, breast cancer survival or overall survival (OS) (1-3). The degree of response depends largely on sensitivity to therapy and is known to vary in the different breast cancer subtypes, where the highest pathological complete response (pCR) rate is reached within the human epidermal growth factor receptor 2 (HER2)-positive and the triple negative subtypes (4-7).

With the neoadjuvant approach, the tumor is left *in situ* during chemotherapy, which enables evaluation of treatment efficacy. Whether pCR is achieved has an impact on patient prognosis, although prognostic value may vary depending on pCR definition and tumor subtype (4). However, the binary pCR measure ignores differences in prognosis within patients with residual disease. For a more comprehensive evaluation of tumor response after NAC, the residual cancer burden (RCB) was therefore developed, which has shown to be prognostic in all phenotypic subtypes of breast cancer (8, 9).

Although important for prognosis, evaluation of the response at NAC is typically only provided in the post-NAC surgical resection specimen, leaving only room for tailoring the treatment post-surgery, i.e. adjuvant therapy. In the optimal situation, reliable information on tumor response is obtained during, or even before start of, NAC treatment providing the opportunity to tailor the neoadjuvant and surgical treatment to the observed tumor response.

Different methods for predicting tumor response prior to surgery are available in daily clinical practice, e.g., physical examination, ultrasound, PET/CT and dynamic contrast enhanced magnetic resonance imaging (DCE-MRI) of the breast. The sensitivity of DCE-MRI for predicting pCR after NAC is reported to range between 65% and 91% and specificity is reported to range from between 81% and 88% (10-12). In clinical practice, these are generally not considered high enough to guide treatment decisions, as missed residual disease and inappropriate adjustment of treatment can have a detrimental effect on patient's prognosis. For instance, if a physician adopts a wait-and-see approach instead of surgery on the basis of complete tumor response at DCE MRI, it may result in undertreatment and early relapse if residual cancer is actually still present in the breast.

A method to improve the accuracy of MRI uses various different imaging protocols in one single session (multiparametric MRI). Hence, the MRI registers information associated with various aspects of tumor biology (proliferation, angiogenesis and metabolism). By adding diffusion weighted imaging (DWI) to the MRI protocol, intratumoral cellularity can be assessed as well, which may improve the value of MRI before, during and after NAC (13, 14).

However, multiparametric MRI is only able to visualize macroscopic disease. To optimize personalized response monitoring, some provision for analysis of microscopic residual disease is needed as well. Repeat core biopsies of the tumor bed during treatment has, however, proven to be hardly feasible in clinical setting(15).

In contrast, liquid biopsies taken from patients' blood are minimally invasive and can contain information from all parts of the tumor, thus potentially capturing intra-tumoral heterogeneity. Liquid biopsies are therefore considered a promising tool for prediction of treatment response(16). Nonetheless, the technique is not yet part of standard clinical practice during NAC. Blood samples of cancer patients can contain circulating tumor cells (CTCs) and circulating DNA. The total of cell-free DNA (cfDNA) can contain DNA from different sources (17). When mutations that are associated with the malignant tumor are found in this

For peer review only - http://bmjopen.bmj.com/site/about/guidelines.xhtml

cfDNA, this is called circulating tumor (ctDNA). Both the total cfDNA and mutations found in ctDNA can contain information on tumor load and tumor biology, which may be of importance for response prediction and prognosis. In patients with breast cancer who are treated with NAC and the presence of CTCs in their blood prior to NAC as well as prior to surgery is associated with worse disease-free survival (DFS) (HR, 2.47; 95% CI; 1.95-3.14) and OS (HR, 2.55; 95% CI 1.91-3.39) (18). In a recent study in triple negative (TN) breast cancer patients treated with NAC, who had residual disease at surgery, an increasing CTC count after surgery was correlated with inferior distant disease free survival (DDFS) (HR, 1.07; 95% CI, 1.01-1.13), DFS (HR, 1.11; 95% CI, 1.03-1.19), and OS (HR, 1.09; 95% CI, 1.02-1.17) (19).

When serial blood samples are taken during treatment, the short half-life of ctDNA (less than 2 hours) allows for changes to be detected quickly and this facilitates dynamic response prediction (20). Tracking of ctDNA mutations during neoadjuvant treatment can give information on presence and load of residual disease as well as associated risk of distant recurrence and mortality (21). ctDNA analysis during treatment may also detect emerging resistance mechanisms, thus allowing the efficacy of anticancer treatments to be monitored (22, 23). Because driver mutations in breast cancer can be present at very low frequencies, especially in early stages of the disease, highly sensitive assays are necessary(24). In addition to mutations, epigenetic changes are also important for cancer evolution. Methylation can also be detected in breast cancer patients' blood samples and have additional prognostic value (25), which may add to more accurate prediction of treatment response. Although literature on the correlation between methylation, prognosis and ctDNA is not as extensive as that for ctDNA and CTC's, one study did show a significantly worse OS rate at 100 months (78% vs. 95%; $p = 0.002$) for breast cancer patients with methylated DNA detected in their blood compared to patients without(26). Another study reported that early clearance of methylated DNA in the blood occurred in breast cancer patients with pCR (n=4), and longer persisting methylated DNA in the blood occurred in patients with partial response (n=17)(27).

In summary, both MRI and liquid biopsies have been assessed individually confirming their potential to be used in response prediction and evaluation of neoadjuvant breast cancer treatment prior to surgery. Little is known about the combined value of these two techniques to improve prediction of response to NAC so that they can guide personalized treatment decisions. One study by Magbanua et. al.(28) found that adding ctDNA information early during treatment to the MRI predictor functional tumor volume (FTV) resulted in a numerical but not statistically significant increase in performance for pCR prediction. The additive value of ctDNA to MRI to predict response to NAC is thus not unequivocally demonstrated, and further research in this field is required. Our study may add to fine-tuning working hypotheses for follow-up studies that may ultimately lead to practical guidelines, as its design allows for easy translation.

Methods

Study objectives

The primary objective is to explore to what extent the combination of multiparametric MRI, and liquid biopsies prior to, during and after completion of NAC, are able to predict residual cancer burden after NAC in addition to conventional clinical and pathological information.

Secondary objective is to use the strategy from the primary objective to predict alternative outcome measures: ypT0 ypN0 (i.e., absence of invasive cancer and *in situ* cancer in the breast and axillary nodes), ypT0/is ypN0 (i.e., absence of invasive cancer in the breast and axillary nodes, irrespective of ductal carcinoma *in situ*), ypT0/is (i.e., absence of invasive cancer in the

For peer review only - http://bmjopen.bmj.com/site/about/guidelines.xhtml

breast irrespective of ductal carcinoma *in situ* or nodal involvement) and residual lesion volume on DCE-MRI following NAC.

Study design

This is a prospective multicenter observational study in breast cancer patients undergoing NAC. The study has been approved by the Medical Ethics Review Committee of the University Medical Center Utrecht (19-396, NL67308.041.19). SPIRIT guidelines were followed(29). In the LIMA study, the complementary expertise of investigators in the MRI and liquid biopsy field have been combined into a consortium. The study participants will be recruited in 4 different Dutch hospitals. Potential study participants are screened by their treating physicians. Written informed consent will be obtained from all participants by their physician or research nurse. All participants will undergo NAC followed by surgery according to the Dutch oncology guidelines (30). Study duration is from diagnosis of invasive breast cancer until the pathological assessment of the resection specimen after surgery. The LIMA study is registered on ClinicalTrials.gov under registration number NCT04223492.

Patient and public involvement

Patients and public were not involved in study design. Results will not be directly disseminated to participating patients because of the unclear clinical relevance to their individual case. Results will be disseminated according to FAIR principles.

Study population

In order to be eligible to participate for the study, a subject must meet all inclusion criteria and none of the exclusion criteria. We aim to include 100 patients.

Inclusion criteria:

Female patients aged 18 years or older

- Histologically proven invasive breast carcinoma
- Planned to receive NAC (and in case of a HER2-positive tumor: addition of trastuzumab and/or pertuzumab)

Exclusion criteria:

- Breast cancer estrogen receptor (ER)-positive and HER2-negative by immunohistochemistry and Bloom and Richardson grade 1
- Inflammatory breast cancer
- Distant metastases on positron emission computed tomography (PET/CT)
- Prior ipsilateral breast cancer (contralateral breast cancer >5 years ago is allowed)
- Other active malignant disease in the past 5 years (excluded squamous cell or basal cell carcinoma of the skin)
- Pregnancy or lactation
- Contra-indications for MRI according to standard hospital guidelines
- Contra-indications for gadolinium-based contrast-agent, including known prior allergic reaction to any contrast-agent, and renal failure, defined by a glomerular filtration rate < 30 mL/min/1.73m^2

Study procedures

For peer review only - http://bmjopen.bmj.com/site/about/guidelines.xhtml

An overview of the study procedures is shown in figure 1. All patients will undergo a PET/CT scan before the start of NAC to ensure no metastases are present at distant sites.

MRI acquisition and analysis

MRI will be performed prior to, during (approximately halfway), and after NAC but before surgery. MRI will take place on 3 Tesla field strength scanners with a standardized scanning protocol. All MRI scans will be centrally revised by an experienced breast radiologist, blinded to predictors and primary outcome. Tumor imaging characteristics including BI-RADS descriptors and tumor dimensions in 3 directions will be recorded in the electronic case report form (eCRF). We will implement robust apparent diffusion coefficient (ADC) mapping using standardization of diffusion weighting factors (b- values). Quantitative imaging features will be extracted automatically from tumor and healthy tissues (reflecting microenvironment). These methods will be developed and extended from previous studies (31). Optionally, the impact of adding PET features and MRI conductivity features may be explored. PET features and MRI conductivity features will be explored/added if a sufficient number of centers is able to provide these features; technical limitations and workflow considerations in hospitals may limit the availability of these additional features.

Liquid biopsies

Blood samples will be taken from the patients before administration of every chemotherapy cycle, and after completion of NAC prior to surgery. Because the optimal time point for liquid biopsy analysis in the neoadjuvant treatment of non-metastatic breast cancer is still unknown, multiple liquid biopsies will be taken at multiple time points over the course of the treatment. This also allows for close monitoring of trends over the course of time.

Blood samples will be drawn into blood collection tubes containing a preservation fluid. The ctDNA blood samples will be centrifuged at a central location and following a standard protocol of 10 minutes at 1600g. They are then stored -80 °C before further processing. Liquid biopsy analyses take place in the lab of Philips in Eindhoven. After transport they are centrifuged at 16000g. All technicians will be blinded to primary and secondary outcome measures, as well as predictors. Every sample has a unique identifier so that technicians are blinded to study participant number and longitudinal order until data collection is completed. For the analysis of the ctDNA a pre-specified mutation- and methylation panel will be used (Supplementary information). We will predominantly rely on a mass spectroscopy system(32). Since mass spectroscopy is not suited to detect copy number variations, we will use digital droplet PCR (ddPCR) to detect ERBB2 amplification (33). The ddPCR method can also be used to detect mutations that are not being picked up by the mass spectroscopy system, and this will be used for PIK3CA mutations (H1047R, E545K, E542K). CTCs will be determined at all time-points. To isolate and analyze CTCs, the blood will be filtered to reduce the amount of candidate cells by a size and compressibility filter step. After staining, the cells are scanned on a slide to identify the cells which meet the criteria to classify as CTC(34, 35).

Pathological evaluation

All pathology review will be centralized at UMC Utrecht and performed by a dedicated breast pathologist with >20 years of experience. Central review will be performed on the pre-NAC needle biopsies and the post-NAC surgical resection specimen. Blinding to results for research

For peer review only - http://bmjopen.bmj.com/site/about/guidelines.xhtml

purposes will be performed, i.e., the researchers that assess the outcome variables (pathology) do not have access to the potential candidate predictors and the other way around.

Diagnostic biopsy Tumor sections will be stained by hematoxylin and eosin (H&E) staining for initial pathology diagnosis including histologic type and grade according to the Nottingham modification of the Bloom and Richardson method(36, 37). Immunohistochemistry staining for tumor markers will be routinely performed on the most representative paraffin block. ER, PR and HER2 will be interpreted according to Dutch guidelines (30). ER and PRs receptor are considered positive if >10% of nuclei stain positive. Tumors with 3+ HER2 score (strong homogeneous membrane staining in >10% of tumor cells) or HER2 gene amplification are considered HER2 positive on central revision.

Surgical resection specimen Management of the resection specimens will be carried out according to the routine clinical protocol. RCB takes the dimensions of the primary tumor bed into account, as well as cellularity, percentage of in situ disease, number of positive lymph nodes and diameter of the largest lymph node metastases. These items will be reviewed in the surgical resection specimen by a trained pathologist. Calculation of the Residual Cancer Burden will be done according to the guidelines and using the calculator provided by the MD Anderson website(38).

Data collection & safety reporting

Treatment regimen and patient characteristics including age, height, weight, menopausal status and AJCC TNM stage(39) will be recorded in the eCRF. For the eCRF a Good Clinical Practice (GCP)-compliant data capture tool will be used, which has direct input validation, edit checks and automatic saving. Personal data will be saved in an encrypted software system with two-factor authentication and limited access for designated study team members only. This study will follow the FAIR principles in handling and storage of data (40). A data safety monitoring board is not implemented because the study is in the negligible-risk category. For this reason, only two adverse events that can be related to the study procedures will be reported as (serious) adverse events: allergic reactions to contrast agents that are administered during the MRI scans and (thrombo)phlebitis as a result of the intravenous catheter. According to regulations, a medical doctor is always present at the MRI unit when contrast is given. Study monitoring is coordinated by the sponsor and bi-annual monitoring visits are planned.

The start date of the study (first patient included) was 2-1-2020 and the expected end date is September 2022.

Statistical analysis plan

A formal sample size and power calculation is impossible for this type of study with a large number of candidate predictor features in relation to the number patients, because meaningful (co-)variance data is lacking to feed informative simulation studies. Nevertheless, similar studies of this size have succeeded in generating clinically meaningful predictive signatures (41). Furthermore, our primary endpoint (RCB) is continuous, increasing the effective sample size compared to a binary outcome (such as pCR). Finally, inclusion of 100 patients is also what we deem feasible based on the number of breast cancer patients treated with NAC in our region in a 2-year time period.

The primary analysis population will include all patients who receive at least one cycle of neo-adjuvant treatment and have the primary outcome assessed (i.e., residual breast cancer burden).

For peer review only - http://bmjopen.bmj.com/site/about/guidelines.xhtml

Patterns of missing data will be inspected and if necessary we will use established methods for multiple imputation to account for missing data under the missing at random (MAR) assumption.
To meet our primary objective we will estimate the over-optimism corrected mean square error and associated 95% confidence intervals for predicting RCB in the primary analysis population using all candidate predictors from the clinical data, biopsy data and imaging data with or without the features from the liquid biopsies. These scenarios are tested at three time points: before, half way through and at the end of NAC treatment. We will use the prediction scenarios with and without liquid biopsies features to examine their additive value to the MRI-clinical-pathology-based model.
To develop the optimal and most parsimonious prediction model for each scenario, we will primarily make use of Least Absolute Shrinkage and Selection Operator (LASSO) penalized linear regression techniques, using bootstrapping to obtain the penalty value that minimizes the mean square error in RCB prediction. This will be repeated in each multiple imputation dataset, and the optimal models from each imputation dataset will then be averaged to obtain one final optimal model for each analyzed scenario. We will repeat all these modelling steps under an additional bootstrap resampling scheme for an additional internal validation step to optimally correct for over-optimism.
Secondary to the estimation of the mean square error of the models, we will assess the models' performance in other ways as well, including: 1) agreement between predicted and actual observed RCB to assess calibration using scatterplots and linear regression analysis; 2) performance of the prediction models when the predictions of RCB as a continuous measure are compared to clinically relevant subgroups of actual RCB using receiver operating curve (ROC) curves (discrimination) and decision curve analysis (net benefit). For our secondary objectives we will use similar data-analysis approaches.

Discussion

With the neoadjuvant approach, the tumor is left *in situ* during chemotherapy. The extent to which the tumor of an individual patients responds to NAC is highly variable. This variability in response means a certain NAC regimen could be overtreatment in one patient, but undertreatment in another. To define the right treatment approach for an individual patients, and to correctly balance the treatment related side-effects and oncological safety, accurate prediction of response is essential. Response prediction could be used to personalize treatment for breast cancer treated with NAC in different scenarios. After completion of NAC, but before surgery, reliable tumor response evaluation is essential for facilitating de-escalation of the surgical treatment of both breast and axilla. If this evaluation is accurate enough, a wait-and-see approach may even be imaginable, sparing patients surgery-associated morbidity.
When response to NAC is assessed at earlier time points during treatment, it can provide a different set of opportunities for tailoring the treatment to individual patients' needs. An inadequate tumor response at interim evaluation may guide the treating physicians to opt for a different (non-cross resistant) chemotherapy regimen, choose a different type of systemic treatment, or adapt (the timing of) surgical intervention. Chemotherapy treatment is associated with comorbidities and reduced quality of life in breast cancer patients. Excellent response at interim evaluation could also be a reason for adapting (the timing of) surgical intervention or

For peer review only - http://bmjopen.bmj.com/site/about/guidelines.xhtml

may make chemotherapy de-escalation possible, thereby sparing patients unnecessary side-effects.

Especially prediction of tumor response before start of any treatment is challenging, but could have a major impact on determining the treatment strategy. Leaving the tumor *in situ* during NAC can carry risks in aggressive tumors that will not respond to NAC. If this (lack of) response to NAC could be reliably predicted beforehand, more effective treatment options may be adopted.

At this point, however, no method for response prediction available in clinical practice is deemed accurate enough to guide this personalized treatment approach. New strategies for predicting response to NAC include image guided tumor bed biopsy for detecting pCR in the breast after NAC in patients with partial or complete radiologic response. Unfortunately, studies have shown relatively high false negative rates ranging from 17.8-37% for detecting pCR (defined as ypT0), which means tumor bed biopsies cannot (yet) be used to safely omit surgery after NAC. This may be explained by the fact that tissue biopsies are prone to sampling error, due to intra-tumoral spatial heterogeneity (42). The invasive nature of tissue biopsies is also a drawback for clinical implementation.

Both multiparametric MRI and liquid biopsies are non-invasive methods for the evaluation of response that are valuable for the prediction of response to NAC. In the LIMA study these techniques are uniquely combined to fully exploit the complementary information they hold.

A study by Magbanua et al. (28) studied the combined use of ctDNA and MRI to predict pCR in patients included in the I-SPY 2 TRIAL (NCT01042379). They found an increase in area under the curve (AUC) by adding ctDNA to an MRI-derived functional tumor volume model after 3 weeks of paclitaxel-based therapy, but the increase did not reach statistical significance. Functional tumor volume and ctDNA both did remain significant predictors of distant recurrence free survival in an exploratory multivariable analysis. Our study may add to these results on several aspects. We opted for a study design that is as close to clinical practice as possible and does not include regular study visits since blood is drawn from the intravenous catheter that is already in place during regular chemotherapy treatment appointments. Our patients are treated according to the most recent standard clinical guidelines. Therefore our study design reflects daily clinical practice, which will add to the generalizability of our findings.

Secondly, the trend that values of liquid biopsy predictors follow between different timepoints may hold important information, apart from these values themselves. Because our study has a liquid biopsy data point at every chemotherapy cycle, meaningful trends can be obtained which could lead to better predictions. Thus, we also account for the fact that the optimal time points and intervals to assess ctDNA in the neoadjuvant setting are currently unknown.

There are a few useful things to consider in translating this study design to a clinical practice situation. Blood samples are analyzed in an external lab which may come with some logistical challenges. Standardized panels will be used for ctDNA analysis. Some breast cancers may not carry any of the mutations in the panel . At this point the frequency of the methylation markers in early-stage breast cancer is unclear, and methylation markers may not be present in all patients. Therefore, a distinction between actual absence of any ctDNA vs. the absence of ctDNA that can be detected by the panels, cannot be made. Additionally, specific patients are excluded: patients with B&R grade 1 hormone receptor positive breast cancer are excluded because of the is the poor NAC treatment results that have been reached for this subtype, and

For peer review only - http://bmjopen.bmj.com/site/about/guidelines.xhtml

the proposed systemic treatment de-escalation prescribed in current guidelines. Patients with inflammatory breast cancer and recent other malignancies are excluded because these could lead to misinterpretation of ctDNA results. Pregnant or lactating women are excluded because their breast tissue on MRI would be influenced too much. Patients with a contra-indication for MRI or contrast are excluded for their safety.

This study is one of the first to combine multiparametric MRI with liquid biopsies to predict response to neoadjuvant chemotherapy in breast cancer. If the results of this study show proof-of-concept for combining these two techniques for accurate response prediction, larger follow-up studies can be designed to validate the value of these combined modalities in daily clinical practice.

For peer review only

Ethics and dissemination

Medical Research Ethics Committee Utrecht has approved this study (NL67308.041.19). Informed consent will be obtained from each participant. All data is anonymized before publication. The findings of this study will be submitted to international peer-reviewed journals.

Word count: 3903

Abbreviations

ADC: apparent diffusion coefficient; AUC: area under the curve; cfDNA: cell-free DNA; CNN: convolutional neural networks; CTC: circulating tumor cell; ctDNA: circulating tumor DNA; DCE: dynamic contrast enhanced; ddPCR: digital droplet polymerase chain reaction; DFS: disease-free survival; DDFS: distant disease free survival; DWI: diffusion weighted imaging; eCRF: electronic case report form; ER: estrogen receptor; FFPE: formalin-fixed paraffin-embedded; FTV: functional tumor volume; GCP: good clinical practice; HER2: human epidermal growth factor receptor 2; H&E: hematoxylin and eosin; LASSO: Least Absolute Shrinkage and Selection Operator; MAR: missing at random; MRI: magnetic resonance imaging; NAC: neoadjuvant chemotherapy; OS: overall survival; pCR: pathological complete response; PET/CT: positron emission computed tomography; PR: progesterone receptor; RCB: residual cancer burden; RFS: recurrence-free survival; ROC: receiver operating curve

Competing interests

The authors declare that they have no competing interests.

Funding

Funding from the European Union Horizon 2020 research and innovation program under grant agreement no. 755333 (LIMA). Funder was not involved in study design.

Authors' contributions

Guarantors of integrity of entire study, L.M.J., K.G.A.G.; study concepts/study design or data acquisition: all authors; future data analysis/interpretation, all authors; future statistical analysis: L.M.J., S.E., K.G.A.G.; manuscript drafting or manuscript revision for important intellectual content, all authors; approval of final version of submitted manuscript, all authors; literature research, L.M.J., P.V.D., E.V.D.W. ; clinical studies, L.M.J., K.G.A.G., P.V.D., E.V.D.W.

Acknowledgements

We would like to acknowledge dr. W.B. Veldhuis for input from a radiological standpoint and we are grateful for the support of the trial bureau of division Imaging&Oncology of the UMC Utrecht, with a special thanks to Shanta Kalaykhan.

This study is funded by European Union Horizon 2020 research and innovation program under grant agreement no. 755333 (LIMA). Other members of this project are employees of Institut national de la santé et de la recherche médicale (INSERM), Montpellier Cancer Institute France, Angle Europe Limited, ALS Automated Lab Solutions GmbH, AGENA Bioscience GmbH, DiaDX, Stilla Technologies, Philips Electronics Nederland B.V. and Philips GmbH.

Figure legend

For peer review only - http://bmjopen.bmj.com/site/about/guidelines.xhtml

Figure 1: schematic overview of the study procedures. All patients undergo an MRI of the breast and a whole body PET/CT before treatment. MRI scans are also performed during and after treatment. Blood samples are collected before every chemotherapy cycle and before surgery.

Literature

1. Asselain B, Barlow W, Bartlett J, Bergh J, Bergsten-Nordström E, Bliss J, et al. Long-term outcomes for neoadjuvant versus adjuvant chemotherapy in early breast cancer: meta-analysis of individual patient data from ten randomised trials. The Lancet Oncology. 2018;19(1):27-39.
2. El Hage Chehade H, Headon H, El Tokhy O, Heeney J, Kasem A, Mokbel K. Is sentinel lymph node biopsy a viable alternative to complete axillary dissection following neoadjuvant chemotherapy in women with node-positive breast cancer at diagnosis? An updated meta-analysis involving 3,398 patients. The American Journal of Surgery. 2016;212(5):969-81.
3. Simons JM, Maaskant-Braat AJG, Luiten EJT, Leidenius MHK, van Nijnatten TJA, Boelens PG, et al. Patterns of axillary staging and management in clinically node positive breast cancer patients treated with neoadjuvant systemic therapy: Results of a survey amongst breast cancer specialists. European Journal of Surgical Oncology. 2020;46(1):53-8.
4. Cortazar P, Zhang L, Untch M, Mehta K, Costantino JP, Wolmark N, et al. Pathological complete response and long-term clinical benefit in breast cancer: the CTNeoBC pooled analysis. Lancet (London, England). 2014;384(9938):164-72.
5. Haque W, Verma V, Hatch S, Suzanne Klimberg V, Brian Butler E, Teh BS. Response rates and pathologic complete response by breast cancer molecular subtype following neoadjuvant chemotherapy. Breast Cancer Res Treat. 2018;170(3):559-67.
6. von Minckwitz G, Untch M, Blohmer JU, Costa SD, Eidtmann H, Fasching PA, et al. Definition and impact of pathologic complete response on prognosis after neoadjuvant chemotherapy in various intrinsic breast cancer subtypes. J Clin Oncol. 2012;30(15):1796-804.
7. Zeidman M, Alberty-Oller JJ, Ru M, Pisapati KV, Moshier E, Ahn S, et al. Use of neoadjuvant versus adjuvant chemotherapy for hormone receptor-positive breast cancer: a National Cancer Database (NCDB) study. Breast Cancer Res Treat. 2020.
8. Symmans WF, Wei C, Gould R, Yu X, Zhang Y, Liu M, et al. Long-Term Prognostic Risk After Neoadjuvant Chemotherapy Associated With Residual Cancer Burden and Breast Cancer Subtype. J Clin Oncol. 2017;35(10):1049-60.
9. Yau C, Van Der Noordaa M, Wei J, Osdoit M, Reyal F, Hamy AS, et al. Residual cancer burden after neoadjuvant therapy and long-term survival outcomes in breast cancer: A multi-center pooled analysis. Cancer Research. 2020;80(4).
10. Liu Q, Wang C, Li P, Liu J, Huang G, Song S. The Role of (18)F-FDG PET/CT and MRI in Assessing Pathological Complete Response to Neoadjuvant Chemotherapy in Patients with Breast Cancer: A Systematic Review and Meta-Analysis. BioMed research international. 2016;2016:3746232.
11. Cheng Q, Huang J, Liang J, Ma M, Ye K, Shi C, et al. The Diagnostic Performance of DCE-MRI in Evaluating the Pathological Response to Neoadjuvant Chemotherapy in Breast Cancer: A Meta-Analysis. Front Oncol. 2020;10:93.
12. Virostko J, Hainline A, Kang H, Arlinghaus LR, Abramson RG, Barnes SL, et al. Dynamic contrast-enhanced magnetic resonance imaging and diffusion-weighted magnetic resonance imaging for predicting the response of locally advanced breast cancer to neoadjuvant therapy: a meta-analysis. Journal of medical imaging (Bellingham, Wash). 2017;5(1):011011.
13. Li W, Newitt DC, Wilmes LJ, Jones EF, Arasu V, Gibbs J, et al. Additive value of diffusion-weighted MRI in the I-SPY 2 TRIAL. J Magn Reson Imaging. 2019.
14. Gu YL, Pan SM, Ren J, Yang ZX, Jiang GQ. Role of Magnetic Resonance Imaging in Detection of Pathologic Complete Remission in Breast Cancer Patients Treated With Neoadjuvant Chemotherapy: A Meta-analysis. Clinical breast cancer. 2017;17(4):245-55.
15. van Loevezijn AA, van der Noordaa MEM, van Werkhoven ED, Loo CE, Winter-Warnars GAO, Wiersma T, et al. Minimally Invasive Complete Response Assessment of the Breast After Neoadjuvant

For peer review only - http://bmjopen.bmj.com/site/about/guidelines.xhtml

Systemic Therapy for Early Breast Cancer (MICRA trial): Interim Analysis of a Multicenter Observational Cohort Study. Ann Surg Oncol. 2021;28(6):3243-53.
16. Jongbloed EM, Deger T, Sleijfer S, Martens JWM, Jager A, Wilting SM. A Systematic Review of the Use of Circulating Cell-Free DNA Dynamics to Monitor Response to Treatment in Metastatic Breast Cancer Patients. Cancers. 2021;13(8):1811.
17. Thierry AR, El Messaoudi S, Gahan PB, Anker P, Stroun M. Origins, structures, and functions of circulating DNA in oncology. Cancer Metastasis Rev. 2016;35(3):347-76.
18. Bidard FC, Michiels S, Riethdorf S, Mueller V, Esserman LJ, Lucci A, et al. Circulating Tumor Cells in Breast Cancer Patients Treated by Neoadjuvant Chemotherapy: A Meta-analysis. Journal of the National Cancer Institute. 2018;110(6):560-7.
19. Radovich M, Jiang G, Hancock BA, Chitambar C, Nanda R, Falkson C, et al. Association of Circulating Tumor DNA and Circulating Tumor Cells After Neoadjuvant Chemotherapy With Disease Recurrence in Patients With Triple-Negative Breast Cancer: Preplanned Secondary Analysis of the BRE12-158 Randomized Clinical Trial. JAMA Oncology. 2020.
20. Diehl F, Schmidt K, Choti MA, Romans K, Goodman S, Li M, et al. Circulating mutant DNA to assess tumor dynamics. Nature Medicine. 2008;14(9):985-90.
21. Riva F, Bidard FC, Houy A, Saliou A, Madic J, Rampanou A, et al. Patient-Specific Circulating Tumor DNA Detection during Neoadjuvant Chemotherapy in Triple-Negative Breast Cancer. Clin Chem. 2017;63(3):691-9.
22. Ma F, Zhu W, Guan Y, Yang L, Xia X, Chen S, et al. ctDNA dynamics: a novel indicator to track resistance in metastatic breast cancer treated with anti-HER2 therapy. Oncotarget. 2016;7(40):66020-31.
23. Hu ZY, Xie N, Tian C, Yang X, Liu L, Li J, et al. Identifying Circulating Tumor DNA Mutation Profiles in Metastatic Breast Cancer Patients with Multiline Resistance. EBioMedicine. 2018;32:111-8.
24. Ignatiadis M, Sledge GW, Jeffrey SS. Liquid biopsy enters the clinic — implementation issues and future challenges. Nature Reviews Clinical Oncology. 2021;18(5):297-312.
25. Panagopoulou M, Karaglani M, Balgkouranidou I, Biziota E, Koukaki T, Karamitrousis E, et al. Circulating cell-free DNA in breast cancer: size profiling, levels, and methylation patterns lead to prognostic and predictive classifiers. Oncogene. 2019.
26. Fujita N, Nakayama T, Yamamoto N, Kim SJ, Shimazu K, Shimomura A, et al. Methylated DNA and total DNA in serum detected by one-step methylation-specific PCR is predictive of poor prognosis for breast cancer patients. Oncology. 2012;83(5):273-82.
27. Avraham A, Uhlmann R, Shperber A, Birnbaum M, Sandbank J, Sella A, et al. Serum DNA methylation for monitoring response to neoadjuvant chemotherapy in breast cancer patients. International journal of cancer. 2012;131(7):E1166-72.
28. Magbanua MJM, Li W, Wolf DM, Yau C, Hirst GL, Swigart LB, et al. Circulating tumor DNA and magnetic resonance imaging to predict neoadjuvant chemotherapy response and recurrence risk. NPJ breast cancer. 2021;7(1):32.
29. Chan A-W, Tetzlaff JM, Gøtzsche PC, Altman DG, Mann H, Berlin JA, et al. SPIRIT 2013 explanation and elaboration: guidance for protocols of clinical trials. BMJ : British Medical Journal. 2013;346:e7586.
30. (IKNL) IkN. Landelijke richtlijn Borstkanker 2020 [Available from: https://richtlijnendatabase.nl.
31. Aerts HJ, Velazquez ER, Leijenaar RT, Parmar C, Grossmann P, Carvalho S, et al. Decoding tumour phenotype by noninvasive imaging using a quantitative radiomics approach. Nat Commun. 2014;5:4006.
32. Mosko MJ, Nakorchevsky AA, Flores E, Metzler H, Ehrich M, van den Boom DJ, et al. Ultrasensitive Detection of Multiplexed Somatic Mutations Using MALDI-TOF Mass Spectrometry. J Mol Diagn. 2016;18(1):23-31.
33. Madic J, Zocevic A, Senlis V, Fradet E, Andre B, Muller S, et al. Three-color crystal digital PCR. Biomol Detect Quantif. 2016;10:34-46.
34. Xu L, Mao X, Imrali A, Syed F, Mutsvangwa K, Berney D, et al. Optimization and Evaluation of a Novel Size Based Circulating Tumor Cell Isolation System. PloS one. 2015;10(9):e0138032.

For peer review only - http://bmjopen.bmj.com/site/about/guidelines.xhtml

35. Neumann MH, Schneck H, Decker Y, Schomer S, Franken A, Endris V, et al. Isolation and characterization of circulating tumor cells using a novel workflow combining the CellSearch((R)) system and the CellCelector(). Biotechnol Prog. 2017;33(1):125-32.
36. Elston CW, Ellis IO. Pathological prognostic factors in breast cancer. I. The value of histological grade in breast cancer: experience from a large study with long-term follow-up. Histopathology. 1991;19(5):403-10.
37. Bloom HJ, Richardson WW. Histological grading and prognosis in breast cancer; a study of 1409 cases of which 359 have been followed for 15 years. Br J Cancer. 1957;11(3):359-77.
38. Center MAC. Residual Cancer Burden Calculator [cited 2018. Available from: http://www3.mdanderson.org/app/medcalc/index.cfm?pagename=jsconvert3.
39. Cancer AJCo. AJCC Cancer Staging Manual, Eight Edition. 2018. p. 622.
40. Wilkinson MD, Dumontier M, Aalbersberg IJ, Appleton G, Axton M, Baak A, et al. The FAIR Guiding Principles for scientific data management and stewardship. Scientific Data. 2016;3(1):160018.
41. van 't Veer LJ, Dai H, van de Vijver MJ, He YD, Hart AA, Mao M, et al. Gene expression profiling predicts clinical outcome of breast cancer. Nature. 2002;415(6871):530-6.
42. Yates LR, Gerstung M, Knappskog S, Desmedt C, Gundem G, Van Loo P, et al. Subclonal diversification of primary breast cancer revealed by multiregion sequencing. Nat Med. 2015;21(7):751-9.

For peer review only

For peer review only - http://bmjopen.bmj.com/site/about/guidelines.xhtml

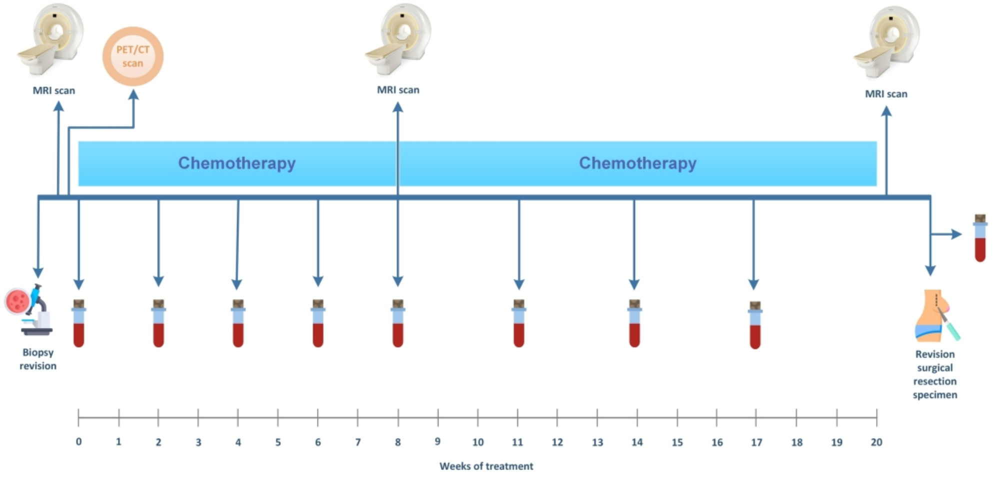

Figure 1: Schematic overview of the study procedures. All patients undergo an MRI of the breast and a whole body PET/CT before treatment. MRI scans are also performed during and after treatment. Blood samples are collected before every chemotherapy cycle and before surgery.

250x120mm (300 x 300 DPI)

Supplementary information

custom UltraSEEK® Breast Cancer Panel[1]	
**Gene**	**Coverage (missense mutations)**
PIK3CA (33.89% freq in invasive breast cancer[2])	N345K, C420R, E542K, E545K, E545Q, E545A, H1047R, H1047L
TP53 (36.34% freq in invasive breast cancer[2])	R175H, R213, Y220C, R248W, R248W, R248Q, R273C, R273H
AKT1 (4.52% freq in invasive breast cancer[2])	E17K, L52R
ERBB2 (1.68% freq in invasive breast cancer[2])	G309E, G309A, S310F, L755R, L755S, L755_T759del, D769H, D769Y, V777L, V777L, L869R
ESR1 (0.63% freq in invasive breast cancer[2])	A283V, K303R, E380Q, V392I, S436P, V534E, L536R, L536Q, Y537N, Y537S, Y537C, D538G, S576L

Breast cfDNA Methylation Panel[3]	
**Gene**	**Genomic location**
AKR1B1	Chr7:134459123
APC	Chr5:112737754
ARHGEF7	Chr13:111115541
BRCA1	Chr17:43125416
COL6A2	Chr21:46098888
GPX7	Chr14:37592244
HIST1H3C	Chr1:52602513
MDGI	Chr17:48578124
RASGRF2	Chr1:13173414
RASSF1A	Chr5:80960894
TM6SF1	Chr3:50340798
FOXA1	Chr5:180591531
SCGB3A1	Chr15:83107646
TMEFF2	Chr2:192194694

1 Giannoudis, A. *et al.* Genomic profiling using the UltraSEEK panel identifies discordancy between paired primary and breast cancer brain metastases and an association with brain metastasis-free survival. *Breast Cancer Res Treat* **190**, 241-253, doi:10.1007/s10549-021-06364-8 (2021).

2 Cerami, E. *et al.* The cBio cancer genomics portal: an open platform for exploring multidimensional cancer genomics data. *Cancer discovery* **2**, 401-404, doi:10.1158/2159-8290.CD-12-0095 (2012).

3 A. Sartori, E. K., D. Irwin, S. Joosse. in *Association for Molecular Pathology 2021* Vol. 23 1567-1649 (Elsevier, 2021).

For peer review only

STANDARD PROTOCOL ITEMS: RECOMMENDATIONS FOR INTERVENTIONAL TRIALS

SPIRIT 2013 Checklist: Recommended items to address in a clinical trial protocol and related documents*

Section/item	ItemNo	Description	Addresed on page number
**Administrative information**			
Title	1	Descriptive title identifying the study design, population, interventions, and, if applicable, trial acronym	1
Trial registration	2a	Trial identifier and registry name. If not yet registered, name of intended registry	1,5
	2b	All items from the World Health Organization Trial Registration Data Set	NA
Protocol version	3	Date and version identifier	NA
Funding	4	Sources and types of financial, material, and other support	11
Roles and responsibilities	5a	Names, affiliations, and roles of protocol contributors	11
	5b	Name and contact information for the trial sponsor	1
	5c	Role of study sponsor and funders, if any, in study design; collection, management, analysis, and interpretation of data; writing of the report; and the decision to submit the report for publication, including whether they will have ultimate authority over any of these activities	11
	5d	Composition, roles, and responsibilities of the coordinating centre, steering committee, endpoint adjudication committee, data management team, and other individuals or groups overseeing the trial, if applicable (see Item 21a for data monitoring committee)	11

For peer review only

**Introduction**			
Background and rationale	6a	Description of research question and justification for undertaking the trial, including summary of relevant studies (published and unpublished) examining benefits and harms for each intervention	3-4
	6b	Explanation for choice of comparators	3-4
Objectives	7	Specific objectives or hypotheses	4
Trial design	8	Description of trial design including type of trial (eg, parallel group, crossover, factorial, single group), allocation ratio, and framework (eg, superiority, equivalence, noninferiority, exploratory)	4-5
**Methods: Participants, interventions, and outcomes**			
Study setting	9	Description of study settings (eg, community clinic, academic hospital) and list of countries where data will be collected. Reference to where list of study sites can be obtained	4-5
Eligibility criteria	10	Inclusion and exclusion criteria for participants. If applicable, eligibility criteria for study centres and individuals who will perform the interventions (eg, surgeons, psychotherapists)	5
Interventions	11a	Interventions for each group with sufficient detail to allow replication, including how and when they will be administered	5-7
	11b	Criteria for discontinuing or modifying allocated interventions for a given trial participant (eg, drug dose change in response to harms, participant request, or improving/worsening disease)	NA
	11c	Strategies to improve adherence to intervention protocols, and any procedures for monitoring adherence (eg, drug tablet return, laboratory tests)	NA
	11d	Relevant concomitant care and interventions that are permitted or prohibited during the trial	NA

For peer review only - http://bmjopen.bmj.com/site/about/guidelines.xhtml

Outcomes	12	Primary, secondary, and other outcomes, including the specific measurement variable (eg, systolic blood pressure), analysis metric (eg, change from baseline, final value, time to event), method of aggregation (eg, median, proportion), and time point for each outcome. Explanation of the clinical relevance of chosen efficacy and harm outcomes is strongly recommended	4
Participant timeline	13	Time schedule of enrolment, interventions (including any run-ins and washouts), assessments, and visits for participants. A schematic diagram is highly recommended (see Figure)	6
Sample size	14	Estimated number of participants needed to achieve study objectives and how it was determined, including clinical and statistical assumptions supporting any sample size calculations	5
Recruitment	15	Strategies for achieving adequate participant enrolment to reach target sample size	4-5
**Methods: Assignment of interventions (for controlled trials)**			
Allocation:			
Sequence generation	16a	Method of generating the allocation sequence (eg, computer-generated random numbers), and list of any factors for stratification. To reduce predictability of a random sequence, details of any planned restriction (eg, blocking) should be provided in a separate document that is unavailable to those who enrol participants or assign interventions	NA
Allocation concealment mechanism	16b	Mechanism of implementing the allocation sequence (eg, central telephone; sequentially numbered, opaque, sealed envelopes), describing any steps to conceal the sequence until interventions are assigned	NA
Implementation	16c	Who will generate the allocation sequence, who will enrol participants, and who will assign participants to interventions	NA
Blinding (masking)	17a	Who will be blinded after assignment to interventions (eg, trial participants, care providers, outcome assessors, data analysts), and how	6-7

For peer review only - http://bmjopen.bmj.com/site/about/guidelines.xhtml

	17b	If blinded, circumstances under which unblinding is permissible, and procedure for revealing a participant's allocated intervention during the trial	NA
**Methods: Data collection, management, and analysis**			
Data collection methods	18a	Plans for assessment and collection of outcome, baseline, and other trial data, including any related processes to promote data quality (eg, duplicate measurements, training of assessors) and a description of study instruments (eg, questionnaires, laboratory tests) along with their reliability and validity, if known. Reference to where data collection forms can be found, if not in the protocol	7
	18b	Plans to promote participant retention and complete follow-up, including list of any outcome data to be collected for participants who discontinue or deviate from intervention protocols	NA
Data management	19	Plans for data entry, coding, security, and storage, including any related processes to promote data quality (eg, double data entry; range checks for data values). Reference to where details of data management procedures can be found, if not in the protocol	7
Statistical methods	20a	Statistical methods for analysing primary and secondary outcomes. Reference to where other details of the statistical analysis plan can be found, if not in the protocol	7-8
	20b	Methods for any additional analyses (eg, subgroup and adjusted analyses)	7-8
	20c	Definition of analysis population relating to protocol non-adherence (eg, as randomised analysis), and any statistical methods to handle missing data (eg, multiple imputation)	7-8
**Methods: Monitoring**			
Data monitoring	21a	Composition of data monitoring committee (DMC); summary of its role and reporting structure; statement of whether it is independent from the sponsor and competing interests; and reference to where further details about its charter can be found, if not in the protocol. Alternatively, an explanation of why a DMC is not needed	7

	21b	Description of any interim analyses and stopping guidelines, including who will have access to these interim results and make the final decision to terminate the trial	NA
Harms	22	Plans for collecting, assessing, reporting, and managing solicited and spontaneously reported adverse events and other unintended effects of trial interventions or trial conduct	7
Auditing	23	Frequency and procedures for auditing trial conduct, if any, and whether the process will be independent from investigators and the sponsor	7
**Ethics and dissemination**			
Research ethics approval	24	Plans for seeking research ethics committee/institutional review board (REC/IRB) approval	4
Protocol amendments	25	Plans for communicating important protocol modifications (eg, changes to eligibility criteria, outcomes, analyses) to relevant parties (eg, investigators, REC/IRBs, trial participants, trial registries, journals, regulators)	4
Consent or assent	26a	Who will obtain informed consent or assent from potential trial participants or authorised surrogates, and how (see Item 32)	5
	26b	Additional consent provisions for collection and use of participant data and biological specimens in ancillary studies, if applicable	5
Confidentiality	27	How personal information about potential and enrolled participants will be collected, shared, and maintained in order to protect confidentiality before, during, and after the trial	7,11
Declaration of interests	28	Financial and other competing interests for principal investigators for the overall trial and each study site	11
Access to data	29	Statement of who will have access to the final trial dataset, and disclosure of contractual agreements that limit such access for investigators	NA

For peer review only

Ancillary and post-trial care	30	Provisions, if any, for ancillary and post-trial care, and for compensation to those who suffer harm from trial participation	NA
Dissemination policy	31a	Plans for investigators and sponsor to communicate trial results to participants, healthcare professionals, the public, and other relevant groups (eg, via publication, reporting in results databases, or other data sharing arrangements), including any publication restrictions	11
	31b	Authorship eligibility guidelines and any intended use of professional writers	NA
	31c	Plans, if any, for granting public access to the full protocol, participant-level dataset, and statistical code	NA
**Appendices**			
Informed consent materials	32	Model consent form and other related documentation given to participants and authorised surrogates	Additional files
Biological specimens	33	Plans for collection, laboratory evaluation, and storage of biological specimens for genetic or molecular analysis in the current trial and for future use in ancillary studies, if applicable	NA

*It is strongly recommended that this checklist be read in conjunction with the SPIRIT 2013 Explanation & Elaboration for important clarification on the items. Amendments to the protocol should be tracked and dated. The SPIRIT checklist is copyrighted by the SPIRIT Group under the Creative Commons "Attribution-NonCommercial-NoDerivs 3.0 Unported" license.

For peer review only

BMJ Open

Improving prediction of response to neoadjuvant treatment in patients with breast cancer by combining liquid biopsies with multiparametric MRI: protocol of the LIMA study, a multicenter prospective observational cohort study

Journal:	*BMJ Open*
Manuscript ID	bmjopen-2022-061334.R2
Article Type:	Protocol
Date Submitted by the Author:	11-Aug-2022
Complete List of Authors:	Janssen, Liselore; University Medical Centre Utrecht, Image Sciences Institute<br>Suelmann, Britt; University Medical Centre Utrecht, Department of Medical Oncology<br>Elias, Sjoerd; University Medical Centre Utrecht, Julius Center for Health Sciences and Primary Care<br>Janse, Markus; University Medical Centre Utrecht, Image Sciences Institute<br>van Diest, P.J.; University Medical Centre Utrecht, Department of Pathology<br>van der Wall, Elsken; University Medical Centre Utrecht, Department of Medical Oncology<br>Gilhuijs, Kenneth; University Medical Centre Utrecht, Image Sciences Institute
**Primary Subject Heading**:	Oncology
Secondary Subject Heading:	Radiology and imaging
Keywords:	Breast tumours < ONCOLOGY, CHEMOTHERAPY, Magnetic resonance imaging < RADIOLOGY & IMAGING, Adult oncology < ONCOLOGY, Breast imaging < RADIOLOGY & IMAGING

SCHOLARONE™ Manuscripts

Title: Improving prediction of response to neoadjuvant treatment in patients with breast cancer by combining liquid biopsies with multiparametric MRI: protocol of the LIMA study, a multicenter prospective observational cohort study

Authors: L.M. Janssen[1], B.B.M. Suelmann[2], S.G. Elias[4], M.H.A. Janse[1], P.J. van Diest[5], E. van der Wall[2], K.G.A. Gilhuijs[1].
Corresponding Author: Kenneth Gilhuijs
Email corresponding author: K.G.A.Gilhuijs@umcutrecht.nl

Affiliations
[1]Image Sciences Institute, University Medical Center Utrecht, Utrecht, the Netherlands.
[2]Department of Medical Oncology, University Medical Center Utrecht, Utrecht University, Utrecht, The Netherlands.
[3]Julius Center for Health Sciences and Primary Care, University Medical Center Utrecht, Utrecht, the Netherlands
[4]Department of Pathology, University Medical Centre Utrecht, PO Box 85500, 3508 GA, Utrecht, The Netherlands

For peer review only

Abstract
Introduction The response to neoadjuvant chemotherapy (NAC) in breast cancer has important prognostic implications. Dynamic prediction of tumor regression by NAC may allow for adaption of the treatment plan before completion, or even before the start of treatment. Such predictions may help prevent overtreatment and related toxicity and correct for undertreatment with ineffective regimens. Current imaging methods are not able to fully predict efficacy of NAC. To successfully improve response prediction, tumor biology and heterogeneity as well as treatment-induced changes have to be considered. In the LIMA study, multiparametric magnetic resonance imaging (MRI) will be combined with liquid biopsies. In addition to conventional clinical and pathological information, these methods may give complementary information at multiple time points during treatment.
Aim: To combine multiparametric MRI and liquid biopsies in breast cancer patients to predict Residual Cancer Burden (RCB) after NAC, in adjunct to standard clinico-pathological information. Predictions will be made before the start of NAC, approximately halfway during treatment and after completion of NAC.
Methods In this multicenter prospective observational study we aim to enroll 100 patients. Multiparametric MRI will be performed prior to NAC, approximately halfway and after completion of NAC. Liquid biopsies will be obtained immediately prior to every cycle of chemotherapy and after completion of NAC. The primary endpoint is RCB in the surgical resection specimen following NAC. Collected data will primarily be analyzed using multivariable techniques such as penalized regression techniques.
Ethics and dissemination Medical Research Ethics Committee Utrecht has approved this study (NL67308.041.19). Informed consent will be obtained from each participant. All data is anonymized before publication. The findings of this study will be submitted to international peer-reviewed journals.
Trial registration
The LIMA study is registered on ClinicalTrials.gov under registration number NCT04223492.

For peer review only - http://bmjopen.bmj.com/site/about/guidelines.xhtml

Strengths and limitations of this study

- The LIMA trial aims to improve prediction of response to neoadjuvant treatment in patients with breast cancer by combining liquid biopsies with multiparametric MRI
- LIMA is a prospective multicenter observational trial that includes women with early stage breast cancer in the Netherlands
- The LIMA trial was designed to resemble daily clinical practice which facilitates translation and adds to generalizability of results
- The LIMA trial has a low burden for recruited patients

Keywords

Breast tumors, chemotherapy, magnetic resonance imaging, adult oncology, breast imaging

For peer review only

Introduction

Neoadjuvant chemotherapy (NAC) has become an important treatment strategy for early stage breast cancer patients. Compared to adjuvant chemotherapy, NAC potentially results in less extensive surgery of both breast and axilla, without compromising distant recurrence, breast cancer survival or overall survival (OS) (1-3). The degree of response depends largely on sensitivity to therapy and is known to vary in the different breast cancer subtypes, where the highest pathological complete response (pCR) rate is reached within the human epidermal growth factor receptor 2 (HER2)-positive and the triple negative subtypes (4-7).

With the neoadjuvant approach, the tumor is left *in situ* during chemotherapy, which enables evaluation of treatment efficacy. Whether pCR is achieved has an impact on patient prognosis, although prognostic value may vary depending on pCR definition and tumor subtype (4). However, the binary pCR measure ignores differences in prognosis within patients with residual disease. For a more comprehensive evaluation of tumor response after NAC, the residual cancer burden (RCB) was therefore developed, which has shown to be prognostic in all phenotypic subtypes of breast cancer (8, 9).

Although important for prognosis, evaluation of the response at NAC is typically only provided in the post-NAC surgical resection specimen, leaving only room for tailoring the treatment post-surgery, i.e. adjuvant therapy. In the optimal situation, reliable information on tumor response is obtained during, or even before start of, NAC treatment providing the opportunity to tailor the neoadjuvant and surgical treatment to the observed tumor response.

Different methods for predicting tumor response prior to surgery are available in daily clinical practice, e.g., physical examination, ultrasound, PET/CT and dynamic contrast enhanced magnetic resonance imaging (DCE-MRI) of the breast. The sensitivity of DCE-MRI for predicting pCR after NAC is reported to range between 65% and 91% and specificity is reported to range from between 81% and 88% (10-12). In clinical practice, these are generally not considered high enough to guide treatment decisions, as missed residual disease and inappropriate adjustment of treatment can have a detrimental effect on patient's prognosis. For instance, if a physician adopts a wait-and-see approach instead of surgery on the basis of complete tumor response at DCE MRI, it may result in undertreatment and early relapse if residual cancer is actually still present in the breast.

A method to improve the accuracy of MRI uses various different imaging protocols in one single session (multiparametric MRI). Hence, the MRI registers information associated with various aspects of tumor biology (proliferation, angiogenesis and metabolism). By adding diffusion weighted imaging (DWI) to the MRI protocol, intratumoral cellularity can be assessed as well, which may improve the value of MRI before, during and after NAC (13, 14).

However, multiparametric MRI is only able to visualize macroscopic disease. To optimize personalized response monitoring, some provision for analysis of microscopic residual disease is needed as well. Repeat core biopsies of the tumor bed during treatment has, however, proven to be hardly feasible in clinical setting(15).

In contrast, liquid biopsies taken from patients' blood are minimally invasive and can contain information from all parts of the tumor, thus potentially capturing intra-tumoral heterogeneity. Liquid biopsies are therefore considered a promising tool for prediction of treatment response(16). Nonetheless, the technique is not yet part of standard clinical practice during NAC. Blood samples of cancer patients can contain circulating tumor cells (CTCs) and circulating DNA. The total of cell-free DNA (cfDNA) can contain DNA from different sources (17). When mutations that are associated with the malignant tumor are found in this

For peer review only - http://bmjopen.bmj.com/site/about/guidelines.xhtml

cfDNA, this is called circulating tumor (ctDNA). Both the total cfDNA and mutations found in ctDNA can contain information on tumor load and tumor biology, which may be of importance for response prediction and prognosis. In patients with breast cancer who are treated with NAC and the presence of CTCs in their blood prior to NAC as well as prior to surgery is associated with worse disease-free survival (DFS) (HR, 2.47; 95% CI; 1.95-3.14) and OS (HR, 2.55; 95% CI 1.91-3.39) (18). In a recent study in triple negative (TN) breast cancer patients treated with NAC, who had residual disease at surgery, an increasing CTC count after surgery was correlated with inferior distant disease free survival (DDFS) (HR, 1.07; 95% CI, 1.01-1.13), DFS (HR, 1.11; 95% CI, 1.03-1.19), and OS (HR, 1.09; 95% CI, 1.02-1.17) (19).

When serial blood samples are taken during treatment, the short half-life of ctDNA (less than 2 hours) allows for changes to be detected quickly and this facilitates dynamic response prediction (20). Tracking of ctDNA mutations during neoadjuvant treatment can give information on presence and load of residual disease as well as associated risk of distant recurrence and mortality (21). ctDNA analysis during treatment may also detect emerging resistance mechanisms, thus allowing the efficacy of anticancer treatments to be monitored (22, 23). Because driver mutations in breast cancer can be present at very low frequencies, especially in early stages of the disease, highly sensitive assays are necessary(24). In addition to mutations, epigenetic changes are also important for cancer evolution. Methylation can also be detected in breast cancer patients' blood samples and have additional prognostic value (25), which may add to more accurate prediction of treatment response. Although literature on the correlation between methylation, prognosis and ctDNA is not as extensive as that for ctDNA and CTC's, one study did show a significantly worse OS rate at 100 months (78% vs. 95%; p = 0.002) for breast cancer patients with methylated DNA detected in their blood compared to patients without(26). Another study reported that early clearance of methylated DNA in the blood occurred in breast cancer patients with pCR (n=4), and longer persisting methylated DNA in the blood occurred in patients with partial response (n=17)(27).

In summary, both MRI and liquid biopsies have been assessed individually confirming their potential to be used in response prediction and evaluation of neoadjuvant breast cancer treatment prior to surgery. Little is known about the combined value of these two techniques to improve prediction of response to NAC so that they can guide personalized treatment decisions. One study by Magbanua et. al.(28) found that adding ctDNA information early during treatment to the MRI predictor functional tumor volume (FTV) resulted in a numerical but not statistically significant increase in performance for pCR prediction. The additive value of ctDNA to MRI to predict response to NAC is thus not unequivocally demonstrated, and further research in this field is required. Our study may add to fine-tuning working hypotheses for follow-up studies that may ultimately lead to practical guidelines, as its design allows for easy translation.

Methods

Study objectives

The primary objective is to explore to what extent the combination of multiparametric MRI, and liquid biopsies prior to, during and after completion of NAC, are able to predict residual cancer burden after NAC in addition to conventional clinical and pathological information.

Secondary objective is to use the strategy from the primary objective to predict alternative outcome measures: ypT0 ypN0 (i.e., absence of invasive cancer and *in situ* cancer in the breast and axillary nodes), ypT0/is ypN0 (i.e., absence of invasive cancer in the breast and axillary nodes, irrespective of ductal carcinoma *in situ*), ypT0/is (i.e., absence of invasive cancer in the

For peer review only - http://bmjopen.bmj.com/site/about/guidelines.xhtml

breast irrespective of ductal carcinoma *in situ* or nodal involvement) and residual lesion volume on DCE-MRI following NAC.

Study design

This is a prospective multicenter observational study in breast cancer patients undergoing NAC. The study has been approved by the Medical Ethics Review Committee of the University Medical Center Utrecht (19-396, NL67308.041.19). SPIRIT guidelines were followed(29). In the LIMA study, the complementary expertise of investigators in the MRI and liquid biopsy field have been combined into a consortium. The study participants will be recruited in 4 different Dutch hospitals. Potential study participants are screened by their treating physicians. Written informed consent will be obtained from all participants by their physician or research nurse. All participants will undergo NAC followed by surgery according to the Dutch oncology guidelines (30). Study duration is from diagnosis of invasive breast cancer until the pathological assessment of the resection specimen after surgery. The LIMA study is registered on ClinicalTrials.gov under registration number NCT04223492.

Patient and public involvement

Patients and public were not involved in study design. Results will not be directly disseminated to participating patients because of the unclear clinical relevance to their individual case. Results will be disseminated according to FAIR principles.

Study population

In order to be eligible to participate for the study, a subject must meet all inclusion criteria and none of the exclusion criteria. We aim to include 100 patients.

Inclusion criteria:

Female patients aged 18 years or older

- Histologically proven invasive breast carcinoma
- Planned to receive NAC (and in case of a HER2-positive tumor: addition of trastuzumab and/or pertuzumab)

Exclusion criteria:

- Breast cancer estrogen receptor (ER)-positive and HER2-negative by immunohistochemistry and Bloom and Richardson grade 1
- Inflammatory breast cancer
- Distant metastases on positron emission computed tomography (PET/CT)
- Prior ipsilateral breast cancer (contralateral breast cancer >5 years ago is allowed)
- Other active malignant disease in the past 5 years (excluded squamous cell or basal cell carcinoma of the skin)
- Pregnancy or lactation
- Contra-indications for MRI according to standard hospital guidelines
- Contra-indications for gadolinium-based contrast-agent, including known prior allergic reaction to any contrast-agent, and renal failure, defined by a glomerular filtration rate < 30 mL/min/1.73m^2

Study procedures

For peer review only

An overview of the study procedures is shown in figure 1. All patients will undergo a PET/CT scan before the start of NAC to ensure no metastases are present at distant sites.

MRI acquisition and analysis

MRI will be performed prior to, during (approximately halfway), and after NAC but before surgery. MRI will take place on 3 Tesla field strength scanners with a standardized scanning protocol. All MRI scans will be centrally revised by an experienced breast radiologist, blinded to predictors and primary outcome. Tumor imaging characteristics including BI-RADS descriptors and tumor dimensions in 3 directions will be recorded in the electronic case report form (eCRF). We will implement robust apparent diffusion coefficient (ADC) mapping using standardization of diffusion weighting factors (b- values). Quantitative imaging features will be extracted automatically from tumor and healthy tissues (reflecting microenvironment). These methods will be developed and extended from previous studies (31). Optionally, the impact of adding PET features and MRI conductivity features may be explored. PET features and MRI conductivity features will be explored/added if >75% of centers is able to provide these features; technical limitations and workflow considerations in hospitals may limit the availability of these additional features.

Liquid biopsies

Blood samples will be taken from the patients before administration of every chemotherapy cycle, and after completion of NAC prior to surgery. Because the optimal time point for liquid biopsy analysis in the neoadjuvant treatment of non-metastatic breast cancer is still unknown, multiple liquid biopsies will be taken at multiple time points over the course of the treatment. This also allows for close monitoring of trends over the course of time.

Blood samples will be drawn into blood collection tubes containing a preservation fluid. The ctDNA blood samples will be centrifuged at a central location and following a standard protocol of 10 minutes at 1600g. They are then stored -80 °C before further processing. Liquid biopsy analyses take place in the lab of Philips in Eindhoven. After transport they are centrifuged at 16000g. All technicians will be blinded to primary and secondary outcome measures, as well as predictors. Every sample has a unique identifier so that technicians are blinded to study participant number and longitudinal order until data collection is completed. For the analysis of the ctDNA a pre-specified mutation- and methylation panel will be used (Supplementary information). We will predominantly rely on a mass spectroscopy system(32). Since mass spectroscopy is not suited to detect copy number variations, we will use digital droplet PCR (ddPCR) to detect ERBB2 amplification (33). The ddPCR method can also be used to detect mutations that are not being picked up by the mass spectroscopy system, and this will be used for PIK3CA mutations (H1047R, E545K, E542K). CTCs will be determined at all time-points. To isolate and analyze CTCs, the blood will be filtered to reduce the amount of candidate cells by a size and compressibility filter step. After staining, the cells are scanned on a slide to identify the cells which meet the criteria to classify as CTC(34, 35).

Pathological evaluation

All pathology review will be centralized at UMC Utrecht and performed by a dedicated breast pathologist with >20 years of experience. Central review will be performed on the pre-NAC needle biopsies and the post-NAC surgical resection specimen. Blinding to results for research

For peer review only - http://bmjopen.bmj.com/site/about/guidelines.xhtml

purposes will be performed, i.e., the researchers that assess the outcome variables (pathology) do not have access to the potential candidate predictors and the other way around.

Diagnostic biopsy Tumor sections will be stained by hematoxylin and eosin (H&E) staining for initial pathology diagnosis including histologic type and grade according to the Nottingham modification of the Bloom and Richardson method(36, 37). Immunohistochemistry staining for tumor markers will be routinely performed on the most representative paraffin block. ER, PR and HER2 will be interpreted according to Dutch guidelines (30). ER and PRs receptor are considered positive if >10% of nuclei stain positive. Tumors with 3+ HER2 score (strong homogeneous membrane staining in >10% of tumor cells) or HER2 gene amplification are considered HER2 positive on central revision.

Surgical resection specimen Management of the resection specimens will be carried out according to the routine clinical protocol. RCB takes the dimensions of the primary tumor bed into account, as well as cellularity, percentage of in situ disease, number of positive lymph nodes and diameter of the largest lymph node metastases. These items will be reviewed in the surgical resection specimen by a trained pathologist. Calculation of the Residual Cancer Burden will be done according to the guidelines and using the calculator provided by the MD Anderson website(38).

Data collection & safety reporting

Treatment regimen and patient characteristics including age, height, weight, menopausal status and AJCC TNM stage(39) will be recorded in the eCRF. For the eCRF a Good Clinical Practice (GCP)-compliant data capture tool will be used, which has direct input validation, edit checks and automatic saving. Personal data will be saved in an encrypted software system with two-factor authentication and limited access for designated study team members only. This study will follow the FAIR principles in handling and storage of data (40). A data safety monitoring board is not implemented because the study is in the negligible-risk category. For this reason, only two adverse events that can be related to the study procedures will be reported as (serious) adverse events: allergic reactions to contrast agents that are administered during the MRI scans and (thrombo)phlebitis as a result of the intravenous catheter. According to regulations, a medical doctor is always present at the MRI unit when contrast is given. Study monitoring is coordinated by the sponsor and bi-annual monitoring visits are planned.

The start date of the study (first patient included) was 2-1-2020 and the expected end date is September 2022.

Statistical analysis plan

A formal sample size and power calculation is impossible for this type of study with a large number of candidate predictor features in relation to the number patients, because meaningful (co-)variance data is lacking to feed informative simulation studies. Nevertheless, similar studies of this size have succeeded in generating clinically meaningful predictive signatures (41). Furthermore, our primary endpoint (RCB) is continuous, increasing the effective sample size compared to a binary outcome (such as pCR). Finally, inclusion of 100 patients is also what we deem feasible based on the number of breast cancer patients treated with NAC in our region in a 2-year time period.

The primary analysis population will include all patients who receive at least one cycle of neo-adjuvant treatment and have the primary outcome assessed (i.e., residual breast cancer burden).

For peer review only - http://bmjopen.bmj.com/site/about/guidelines.xhtml

Patterns of missing data will be inspected and if necessary we will use established methods for multiple imputation to account for missing data under the missing at random (MAR) assumption.

To meet our primary objective we will estimate the over-optimism corrected mean square error and associated 95% confidence intervals for predicting RCB in the primary analysis population using all candidate predictors from the clinical data, biopsy data and imaging data with or without the features from the liquid biopsies. These scenarios are tested at three time points: before, half way through and at the end of NAC treatment. We will use the prediction scenarios with and without liquid biopsies features to examine their additive value to the MRI-clinical-pathology-based model.

To develop the optimal and most parsimonious prediction model for each scenario, we will primarily make use of Least Absolute Shrinkage and Selection Operator (LASSO) penalized linear regression techniques, using bootstrapping to obtain the penalty value that minimizes the mean square error in RCB prediction. This will be repeated in each multiple imputation dataset, and the optimal models from each imputation dataset will then be averaged to obtain one final optimal model for each analyzed scenario. We will repeat all these modelling steps under an additional bootstrap resampling scheme for an additional internal validation step to optimally correct for over-optimism.

Secondary to the estimation of the mean square error of the models, we will assess the models' performance in other ways as well, including: 1) agreement between predicted and actual observed RCB to assess calibration using scatterplots and linear regression analysis; 2) performance of the prediction models when the predictions of RCB as a continuous measure are compared to clinically relevant subgroups of actual RCB using receiver operating curve (ROC) curves (discrimination) and decision curve analysis (net benefit). For our secondary objectives we will use similar data-analysis approaches.

Discussion

With the neoadjuvant approach, the tumor is left *in situ* during chemotherapy. The extent to which the tumor of an individual patients responds to NAC is highly variable. This variability in response means a certain NAC regimen could be overtreatment in one patient, but undertreatment in another. To define the right treatment approach for an individual patients, and to correctly balance the treatment related side-effects and oncological safety, accurate prediction of response is essential. Response prediction could be used to personalize treatment for breast cancer treated with NAC in different scenarios. After completion of NAC, but before surgery, reliable tumor response evaluation is essential for facilitating de-escalation of the surgical treatment of both breast and axilla. If this evaluation is accurate enough, a wait-and-see approach may even be imaginable, sparing patients surgery-associated morbidity.

When response to NAC is assessed at earlier time points during treatment, it can provide a different set of opportunities for tailoring the treatment to individual patients' needs. An inadequate tumor response at interim evaluation may guide the treating physicians to opt for a different (non-cross resistant) chemotherapy regimen, choose a different type of systemic treatment, or adapt (the timing of) surgical intervention. Chemotherapy treatment is associated with comorbidities and reduced quality of life in breast cancer patients. Excellent response at interim evaluation could also be a reason for adapting (the timing of) surgical intervention or

For peer review only - http://bmjopen.bmj.com/site/about/guidelines.xhtml

may make chemotherapy de-escalation possible, thereby sparing patients unnecessary side-effects.

Especially prediction of tumor response before start of any treatment is challenging, but could have a major impact on determining the treatment strategy. Leaving the tumor *in situ* during NAC can carry risks in aggressive tumors that will not respond to NAC. If this (lack of) response to NAC could be reliably predicted beforehand, more effective treatment options may be adopted.

At this point, however, no method for response prediction available in clinical practice is deemed accurate enough to guide this personalized treatment approach. New strategies for predicting response to NAC include image guided tumor bed biopsy for detecting pCR in the breast after NAC in patients with partial or complete radiologic response. Unfortunately, studies have shown relatively high false negative rates ranging from 17.8-37% for detecting pCR (defined as ypT0), which means tumor bed biopsies cannot (yet) be used to safely omit surgery after NAC. This may be explained by the fact that tissue biopsies are prone to sampling error, due to intra-tumoral spatial heterogeneity (42). The invasive nature of tissue biopsies is also a drawback for clinical implementation.

Both multiparametric MRI and liquid biopsies are non-invasive methods for the evaluation of response that are valuable for the prediction of response to NAC. In the LIMA study these techniques are uniquely combined to fully exploit the complementary information they hold.

A study by Magbanua et al. (28) studied the combined use of ctDNA and MRI to predict pCR in patients included in the I-SPY 2 TRIAL (NCT01042379). They found an increase in area under the curve (AUC) by adding ctDNA to an MRI-derived functional tumor volume model after 3 weeks of paclitaxel-based therapy, but the increase did not reach statistical significance. Functional tumor volume and ctDNA both did remain significant predictors of distant recurrence free survival in an exploratory multivariable analysis. Our study may add to these results on several aspects. We opted for a study design that is as close to clinical practice as possible and does not include regular study visits since blood is drawn from the intravenous catheter that is already in place during regular chemotherapy treatment appointments. Our patients are treated according to the most recent standard clinical guidelines. Therefore our study design reflects daily clinical practice, which will add to the generalizability of our findings.

Secondly, the trend that values of liquid biopsy predictors follow between different timepoints may hold important information, apart from these values themselves. Because our study has a liquid biopsy data point at every chemotherapy cycle, meaningful trends can be obtained which could lead to better predictions. Thus, we also account for the fact that the optimal time points and intervals to assess ctDNA in the neoadjuvant setting are currently unknown.

There are a few useful things to consider in translating this study design to a clinical practice situation. Blood samples are analyzed in an external lab which may come with some logistical challenges. Standardized panels will be used for ctDNA analysis. Some breast cancers may not carry any of the mutations in the panel . At this point the frequency of the methylation markers in early-stage breast cancer is unclear, and methylation markers may not be present in all patients. Therefore, a distinction between actual absence of any ctDNA vs. the absence of ctDNA that can be detected by the panels, cannot be made. Additionally, specific patients are excluded: patients with B&R grade 1 hormone receptor positive breast cancer are excluded because of the is the poor NAC treatment results that have been reached for this subtype, and

For peer review only - http://bmjopen.bmj.com/site/about/guidelines.xhtml

the proposed systemic treatment de-escalation prescribed in current guidelines. Patients with inflammatory breast cancer and recent other malignancies are excluded because these could lead to misinterpretation of ctDNA results. Pregnant or lactating women are excluded because their breast tissue on MRI would be influenced too much. Patients with a contra-indication for MRI or contrast are excluded for their safety.

This study is one of the first to combine multiparametric MRI with liquid biopsies to predict response to neoadjuvant chemotherapy in breast cancer. If the results of this study show proof-of-concept for combining these two techniques for accurate response prediction, larger follow-up studies can be designed to validate the value of these combined modalities in daily clinical practice.

For peer review only

Ethics and dissemination

Medical Research Ethics Committee Utrecht has approved this study (NL67308.041.19). Informed consent will be obtained from each participant. All data is anonymized before publication. The findings of this study will be submitted to international peer-reviewed journals.

Word count: 3903

Abbreviations

ADC: apparent diffusion coefficient; AUC: area under the curve; cfDNA: cell-free DNA; CNN: convolutional neural networks; CTC: circulating tumor cell; ctDNA: circulating tumor DNA; DCE: dynamic contrast enhanced; ddPCR: digital droplet polymerase chain reaction; DFS: disease-free survival; DDFS: distant disease free survival; DWI: diffusion weighted imaging; eCRF: electronic case report form; ER: estrogen receptor; FFPE: formalin-fixed paraffin-embedded; FTV: functional tumor volume; GCP: good clinical practice; HER2: human epidermal growth factor receptor 2; H&E: hematoxylin and eosin; LASSO: Least Absolute Shrinkage and Selection Operator; MAR: missing at random; MRI: magnetic resonance imaging; NAC: neoadjuvant chemotherapy; OS: overall survival; pCR: pathological complete response; PET/CT: positron emission computed tomography; PR: progesterone receptor; RCB: residual cancer burden; RFS: recurrence-free survival; ROC: receiver operating curve

Competing interests

The authors declare that they have no competing interests.

Funding

Funding from the European Union Horizon 2020 research and innovation program under grant agreement no. 755333 (LIMA). Funder was not involved in study design.

Authors' contributions

KG and EW conceived the study; KG is the principal investigator of the grant; LJ, EW, SE and KG designed the final study protocol; PD and BS helped in the design of the final study protocol; LJ and KG coordinated ethics approval; LJ coordinated collaboration among investigators from all institutions; PD, EW and KG provided the domain knowledge expertise; MJ contributed to the technical design; SE provided biostatistical and epidemiological support; LJ, PD, SB and EW provided clinical input and perspectives to the qualitative aspects of the study; LJ drafted the initial manuscript; EW, KG, and SE revised the initial manuscript draft; All authors read and approved the final.

Acknowledgements

We would like to acknowledge dr. W.B. Veldhuis for input from a radiological standpoint and we are grateful for the support of the trial bureau of division Imaging&Oncology of the UMC Utrecht, with a special thanks to Shanta Kalaykhan.

This study is funded by European Union Horizon 2020 research and innovation program under grant agreement no. 755333 (LIMA). Other members of this project are employees of Institut national de la santé et de la recherche médicale (INSERM), Montpellier Cancer Institute France, Angle Europe Limited, ALS Automated Lab Solutions GmbH, AGENA Bioscience GmbH, DiaDX, Stilla Technologies, Philips Electronics Nederland B.V. and Philips GmbH.

For peer review only - http://bmjopen.bmj.com/site/about/guidelines.xhtml

Figure legend

Figure 1: schematic overview of the study procedures. All patients undergo an MRI of the breast and a whole body PET/CT before treatment. MRI scans are also performed during and after treatment. Blood samples are collected before every chemotherapy cycle and before surgery.

Literature

1. Asselain B, Barlow W, Bartlett J, Bergh J, Bergsten-Nordström E, Bliss J, et al. Long-term outcomes for neoadjuvant versus adjuvant chemotherapy in early breast cancer: meta-analysis of individual patient data from ten randomised trials. The Lancet Oncology. 2018;19(1):27-39.
2. El Hage Chehade H, Headon H, El Tokhy O, Heeney J, Kasem A, Mokbel K. Is sentinel lymph node biopsy a viable alternative to complete axillary dissection following neoadjuvant chemotherapy in women with node-positive breast cancer at diagnosis? An updated meta-analysis involving 3,398 patients. The American Journal of Surgery. 2016;212(5):969-81.
3. Simons JM, Maaskant-Braat AJG, Luiten EJT, Leidenius MHK, van Nijnatten TJA, Boelens PG, et al. Patterns of axillary staging and management in clinically node positive breast cancer patients treated with neoadjuvant systemic therapy: Results of a survey amongst breast cancer specialists. European Journal of Surgical Oncology. 2020;46(1):53-8.
4. Cortazar P, Zhang L, Untch M, Mehta K, Costantino JP, Wolmark N, et al. Pathological complete response and long-term clinical benefit in breast cancer: the CTNeoBC pooled analysis. Lancet (London, England). 2014;384(9938):164-72.
5. Haque W, Verma V, Hatch S, Suzanne Klimberg V, Brian Butler E, Teh BS. Response rates and pathologic complete response by breast cancer molecular subtype following neoadjuvant chemotherapy. Breast Cancer Res Treat. 2018;170(3):559-67.
6. von Minckwitz G, Untch M, Blohmer JU, Costa SD, Eidtmann H, Fasching PA, et al. Definition and impact of pathologic complete response on prognosis after neoadjuvant chemotherapy in various intrinsic breast cancer subtypes. J Clin Oncol. 2012;30(15):1796-804.
7. Zeidman M, Alberty-Oller JJ, Ru M, Pisapati KV, Moshier E, Ahn S, et al. Use of neoadjuvant versus adjuvant chemotherapy for hormone receptor-positive breast cancer: a National Cancer Database (NCDB) study. Breast Cancer Res Treat. 2020.
8. Symmans WF, Wei C, Gould R, Yu X, Zhang Y, Liu M, et al. Long-Term Prognostic Risk After Neoadjuvant Chemotherapy Associated With Residual Cancer Burden and Breast Cancer Subtype. J Clin Oncol. 2017;35(10):1049-60.
9. Yau C, Van Der Noordaa M, Wei J, Osdoit M, Reyal F, Hamy AS, et al. Residual cancer burden after neoadjuvant therapy and long-term survival outcomes in breast cancer: A multi-center pooled analysis. Cancer Research. 2020;80(4).
10. Liu Q, Wang C, Li P, Liu J, Huang G, Song S. The Role of (18)F-FDG PET/CT and MRI in Assessing Pathological Complete Response to Neoadjuvant Chemotherapy in Patients with Breast Cancer: A Systematic Review and Meta-Analysis. BioMed research international. 2016;2016:3746232.
11. Cheng Q, Huang J, Liang J, Ma M, Ye K, Shi C, et al. The Diagnostic Performance of DCE-MRI in Evaluating the Pathological Response to Neoadjuvant Chemotherapy in Breast Cancer: A Meta-Analysis. Front Oncol. 2020;10:93.
12. Virostko J, Hainline A, Kang H, Arlinghaus LR, Abramson RG, Barnes SL, et al. Dynamic contrast-enhanced magnetic resonance imaging and diffusion-weighted magnetic resonance imaging for predicting the response of locally advanced breast cancer to neoadjuvant therapy: a meta-analysis. Journal of medical imaging (Bellingham, Wash). 2017;5(1):011011.
13. Li W, Newitt DC, Wilmes LJ, Jones EF, Arasu V, Gibbs J, et al. Additive value of diffusion-weighted MRI in the I-SPY 2 TRIAL. J Magn Reson Imaging. 2019.
14. Gu YL, Pan SM, Ren J, Yang ZX, Jiang GQ. Role of Magnetic Resonance Imaging in Detection of Pathologic Complete Remission in Breast Cancer Patients Treated With Neoadjuvant Chemotherapy: A Meta-analysis. Clinical breast cancer. 2017;17(4):245-55.

For peer review only - http://bmjopen.bmj.com/site/about/guidelines.xhtml

15. van Loevezijn AA, van der Noordaa MEM, van Werkhoven ED, Loo CE, Winter-Warnars GAO, Wiersma T, et al. Minimally Invasive Complete Response Assessment of the Breast After Neoadjuvant Systemic Therapy for Early Breast Cancer (MICRA trial): Interim Analysis of a Multicenter Observational Cohort Study. Ann Surg Oncol. 2021;28(6):3243-53.
16. Jongbloed EM, Deger T, Sleijfer S, Martens JWM, Jager A, Wilting SM. A Systematic Review of the Use of Circulating Cell-Free DNA Dynamics to Monitor Response to Treatment in Metastatic Breast Cancer Patients. Cancers. 2021;13(8):1811.
17. Thierry AR, El Messaoudi S, Gahan PB, Anker P, Stroun M. Origins, structures, and functions of circulating DNA in oncology. Cancer Metastasis Rev. 2016;35(3):347-76.
18. Bidard FC, Michiels S, Riethdorf S, Mueller V, Esserman LJ, Lucci A, et al. Circulating Tumor Cells in Breast Cancer Patients Treated by Neoadjuvant Chemotherapy: A Meta-analysis. Journal of the National Cancer Institute. 2018;110(6):560-7.
19. Radovich M, Jiang G, Hancock BA, Chitambar C, Nanda R, Falkson C, et al. Association of Circulating Tumor DNA and Circulating Tumor Cells After Neoadjuvant Chemotherapy With Disease Recurrence in Patients With Triple-Negative Breast Cancer: Preplanned Secondary Analysis of the BRE12-158 Randomized Clinical Trial. JAMA Oncology. 2020.
20. Diehl F, Schmidt K, Choti MA, Romans K, Goodman S, Li M, et al. Circulating mutant DNA to assess tumor dynamics. Nature Medicine. 2008;14(9):985-90.
21. Riva F, Bidard FC, Houy A, Saliou A, Madic J, Rampanou A, et al. Patient-Specific Circulating Tumor DNA Detection during Neoadjuvant Chemotherapy in Triple-Negative Breast Cancer. Clin Chem. 2017;63(3):691-9.
22. Ma F, Zhu W, Guan Y, Yang L, Xia X, Chen S, et al. ctDNA dynamics: a novel indicator to track resistance in metastatic breast cancer treated with anti-HER2 therapy. Oncotarget. 2016;7(40):66020-31.
23. Hu ZY, Xie N, Tian C, Yang X, Liu L, Li J, et al. Identifying Circulating Tumor DNA Mutation Profiles in Metastatic Breast Cancer Patients with Multiline Resistance. EBioMedicine. 2018;32:111-8.
24. Ignatiadis M, Sledge GW, Jeffrey SS. Liquid biopsy enters the clinic — implementation issues and future challenges. Nature Reviews Clinical Oncology. 2021;18(5):297-312.
25. Panagopoulou M, Karaglani M, Balgkouranidou I, Biziota E, Koukaki T, Karamitrousis E, et al. Circulating cell-free DNA in breast cancer: size profiling, levels, and methylation patterns lead to prognostic and predictive classifiers. Oncogene. 2019.
26. Fujita N, Nakayama T, Yamamoto N, Kim SJ, Shimazu K, Shimomura A, et al. Methylated DNA and total DNA in serum detected by one-step methylation-specific PCR is predictive of poor prognosis for breast cancer patients. Oncology. 2012;83(5):273-82.
27. Avraham A, Uhlmann R, Shperber A, Birnbaum M, Sandbank J, Sella A, et al. Serum DNA methylation for monitoring response to neoadjuvant chemotherapy in breast cancer patients. International journal of cancer. 2012;131(7):E1166-72.
28. Magbanua MJM, Li W, Wolf DM, Yau C, Hirst GL, Swigart LB, et al. Circulating tumor DNA and magnetic resonance imaging to predict neoadjuvant chemotherapy response and recurrence risk. NPJ breast cancer. 2021;7(1):32.
29. Chan A-W, Tetzlaff JM, Gøtzsche PC, Altman DG, Mann H, Berlin JA, et al. SPIRIT 2013 explanation and elaboration: guidance for protocols of clinical trials. BMJ : British Medical Journal. 2013;346:e7586.
30. (IKNL) IkN. Landelijke richtlijn Borstkanker 2020 [Available from: https://richtlijnendatabase.nl.
31. Aerts HJ, Velazquez ER, Leijenaar RT, Parmar C, Grossmann P, Carvalho S, et al. Decoding tumour phenotype by noninvasive imaging using a quantitative radiomics approach. Nat Commun. 2014;5:4006.
32. Mosko MJ, Nakorchevsky AA, Flores E, Metzler H, Ehrich M, van den Boom DJ, et al. Ultrasensitive Detection of Multiplexed Somatic Mutations Using MALDI-TOF Mass Spectrometry. J Mol Diagn. 2016;18(1):23-31.
33. Madic J, Zocevic A, Senlis V, Fradet E, Andre B, Muller S, et al. Three-color crystal digital PCR. Biomol Detect Quantif. 2016;10:34-46.

For peer review only - http://bmjopen.bmj.com/site/about/guidelines.xhtml

34. Xu L, Mao X, Imrali A, Syed F, Mutsvangwa K, Berney D, et al. Optimization and Evaluation of a Novel Size Based Circulating Tumor Cell Isolation System. PloS one. 2015;10(9):e0138032.
35. Neumann MH, Schneck H, Decker Y, Schomer S, Franken A, Endris V, et al. Isolation and characterization of circulating tumor cells using a novel workflow combining the CellSearch((R)) system and the CellCelector(). Biotechnol Prog. 2017;33(1):125-32.
36. Elston CW, Ellis IO. Pathological prognostic factors in breast cancer. I. The value of histological grade in breast cancer: experience from a large study with long-term follow-up. Histopathology. 1991;19(5):403-10.
37. Bloom HJ, Richardson WW. Histological grading and prognosis in breast cancer; a study of 1409 cases of which 359 have been followed for 15 years. Br J Cancer. 1957;11(3):359-77.
38. Center MAC. Residual Cancer Burden Calculator [cited 2018. Available from: http://www3.mdanderson.org/app/medcalc/index.cfm?pagename=jsconvert3.
39. Cancer AJCo. AJCC Cancer Staging Manual, Eight Edition. 2018. p. 622.
40. Wilkinson MD, Dumontier M, Aalbersberg IJ, Appleton G, Axton M, Baak A, et al. The FAIR Guiding Principles for scientific data management and stewardship. Scientific Data. 2016;3(1):160018.
41. van 't Veer LJ, Dai H, van de Vijver MJ, He YD, Hart AA, Mao M, et al. Gene expression profiling predicts clinical outcome of breast cancer. Nature. 2002;415(6871):530-6.
42. Yates LR, Gerstung M, Knappskog S, Desmedt C, Gundem G, Van Loo P, et al. Subclonal diversification of primary breast cancer revealed by multiregion sequencing. Nat Med. 2015;21(7):751-9.

For peer review only

For peer review only - http://bmjopen.bmj.com/site/about/guidelines.xhtml

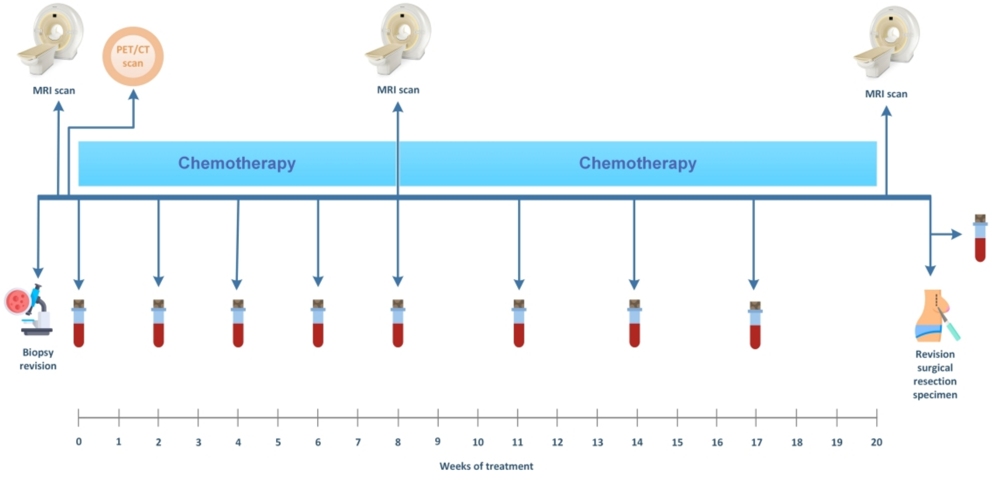

Figure 1: Schematic overview of the study procedures. All patients undergo an MRI of the breast and a whole body PET/CT before treatment. MRI scans are also performed during and after treatment. Blood samples are collected before every chemotherapy cycle and before surgery.

250x120mm (600 x 600 DPI)

Supplementary information

custom UltraSEEK® Breast Cancer Panel[1]	
**Gene**	**Coverage (missense mutations)**
PIK3CA (33.89% freq in invasive breast cancer[2])	N345K, C420R, E542K, E545K, E545Q, E545A, H1047R, H1047L
TP53 (36.34% freq in invasive breast cancer[2])	R175H, R213, Y220C, R248W, R248W, R248Q, R273C, R273H
AKT1 (4.52% freq in invasive breast cancer[2])	E17K, L52R
ERBB2 (1.68% freq in invasive breast cancer[2])	G309E, G309A, S310F, L755R, L755S, L755_T759del, D769H, D769Y, V777L, V777L, L869R
ESR1 (0.63% freq in invasive breast cancer[2])	A283V, K303R, E380Q, V392I, S436P, V534E, L536R, L536Q, Y537N, Y537S, Y537C, D538G, S576L

Breast cfDNA Methylation Panel[3]	
**Gene**	**Genomic location**
AKR1B1	Chr7:134459123
APC	Chr5:112737754
ARHGEF7	Chr13:111115541
BRCA1	Chr17:43125416
COL6A2	Chr21:46098888
GPX7	Chr14:37592244
HIST1H3C	Chr1:52602513
MDGI	Chr17:48578124
RASGRF2	Chr1:13173414
RASSF1A	Chr5:80960894
TM6SF1	Chr3:50340798
FOXA1	Chr5:180591531
SCGB3A1	Chr15:83107646
TMEFF2	Chr2:192194694

For peer review only

1 Giannoudis, A. *et al.* Genomic profiling using the UltraSEEK panel identifies discordancy between paired primary and breast cancer brain metastases and an association with brain metastasis-free survival. *Breast Cancer Res Treat* **190**, 241-253, doi:10.1007/s10549-021-06364-8 (2021).

2 Cerami, E. *et al.* The cBio cancer genomics portal: an open platform for exploring multidimensional cancer genomics data. *Cancer discovery* **2**, 401-404, doi:10.1158/2159-8290.CD-12-0095 (2012).

3 A. Sartori, E. K., D. Irwin, S. Joosse. in *Association for Molecular Pathology 2021* Vol. 23 1567-1649 (Elsevier, 2021).

STANDARD PROTOCOL ITEMS: RECOMMENDATIONS FOR INTERVENTIONAL TRIALS

SPIRIT 2013 Checklist: Recommended items to address in a clinical trial protocol and related documents*

Section/item	ItemNo	Description	Addresed on page number
**Administrative information**			
Title	1	Descriptive title identifying the study design, population, interventions, and, if applicable, trial acronym	1
Trial registration	2a	Trial identifier and registry name. If not yet registered, name of intended registry	1,5
	2b	All items from the World Health Organization Trial Registration Data Set	NA
Protocol version	3	Date and version identifier	NA
Funding	4	Sources and types of financial, material, and other support	11
Roles and responsibilities	5a	Names, affiliations, and roles of protocol contributors	11
	5b	Name and contact information for the trial sponsor	1
	5c	Role of study sponsor and funders, if any, in study design; collection, management, analysis, and interpretation of data; writing of the report; and the decision to submit the report for publication, including whether they will have ultimate authority over any of these activities	11
	5d	Composition, roles, and responsibilities of the coordinating centre, steering committee, endpoint adjudication committee, data management team, and other individuals or groups overseeing the trial, if applicable (see Item 21a for data monitoring committee)	11

For peer review only

Introduction

Background and rationale	6a	Description of research question and justification for undertaking the trial, including summary of relevant studies (published and unpublished) examining benefits and harms for each intervention	3-4
	6b	Explanation for choice of comparators	3-4
Objectives	7	Specific objectives or hypotheses	4
Trial design	8	Description of trial design including type of trial (eg, parallel group, crossover, factorial, single group), allocation ratio, and framework (eg, superiority, equivalence, noninferiority, exploratory)	4-5

Methods: Participants, interventions, and outcomes

Study setting	9	Description of study settings (eg, community clinic, academic hospital) and list of countries where data will be collected. Reference to where list of study sites can be obtained	4-5
Eligibility criteria	10	Inclusion and exclusion criteria for participants. If applicable, eligibility criteria for study centres and individuals who will perform the interventions (eg, surgeons, psychotherapists)	5
Interventions	11a	Interventions for each group with sufficient detail to allow replication, including how and when they will be administered	5-7
	11b	Criteria for discontinuing or modifying allocated interventions for a given trial participant (eg, drug dose change in response to harms, participant request, or improving/worsening disease)	NA
	11c	Strategies to improve adherence to intervention protocols, and any procedures for monitoring adherence (eg, drug tablet return, laboratory tests)	NA
	11d	Relevant concomitant care and interventions that are permitted or prohibited during the trial	NA

For peer review only - http://bmjopen.bmj.com/site/about/guidelines.xhtml

Outcomes	12	Primary, secondary, and other outcomes, including the specific measurement variable (eg, systolic blood pressure), analysis metric (eg, change from baseline, final value, time to event), method of aggregation (eg, median, proportion), and time point for each outcome. Explanation of the clinical relevance of chosen efficacy and harm outcomes is strongly recommended	4
Participant timeline	13	Time schedule of enrolment, interventions (including any run-ins and washouts), assessments, and visits for participants. A schematic diagram is highly recommended (see Figure)	6
Sample size	14	Estimated number of participants needed to achieve study objectives and how it was determined, including clinical and statistical assumptions supporting any sample size calculations	5
Recruitment	15	Strategies for achieving adequate participant enrolment to reach target sample size	4-5
**Methods: Assignment of interventions (for controlled trials)**			
Allocation:			
Sequence generation	16a	Method of generating the allocation sequence (eg, computer-generated random numbers), and list of any factors for stratification. To reduce predictability of a random sequence, details of any planned restriction (eg, blocking) should be provided in a separate document that is unavailable to those who enrol participants or assign interventions	NA
Allocation concealment mechanism	16b	Mechanism of implementing the allocation sequence (eg, central telephone; sequentially numbered, opaque, sealed envelopes), describing any steps to conceal the sequence until interventions are assigned	NA
Implementation	16c	Who will generate the allocation sequence, who will enrol participants, and who will assign participants to interventions	NA
Blinding (masking)	17a	Who will be blinded after assignment to interventions (eg, trial participants, care providers, outcome assessors, data analysts), and how	6-7

	17b	If blinded, circumstances under which unblinding is permissible, and procedure for revealing a participant's allocated intervention during the trial	NA
**Methods: Data collection, management, and analysis**			
Data collection methods	18a	Plans for assessment and collection of outcome, baseline, and other trial data, including any related processes to promote data quality (eg, duplicate measurements, training of assessors) and a description of study instruments (eg, questionnaires, laboratory tests) along with their reliability and validity, if known. Reference to where data collection forms can be found, if not in the protocol	7
	18b	Plans to promote participant retention and complete follow-up, including list of any outcome data to be collected for participants who discontinue or deviate from intervention protocols	NA
Data management	19	Plans for data entry, coding, security, and storage, including any related processes to promote data quality (eg, double data entry; range checks for data values). Reference to where details of data management procedures can be found, if not in the protocol	7
Statistical methods	20a	Statistical methods for analysing primary and secondary outcomes. Reference to where other details of the statistical analysis plan can be found, if not in the protocol	7-8
	20b	Methods for any additional analyses (eg, subgroup and adjusted analyses)	7-8
	20c	Definition of analysis population relating to protocol non-adherence (eg, as randomised analysis), and any statistical methods to handle missing data (eg, multiple imputation)	7-8
**Methods: Monitoring**			
Data monitoring	21a	Composition of data monitoring committee (DMC); summary of its role and reporting structure; statement of whether it is independent from the sponsor and competing interests; and reference to where further details about its charter can be found, if not in the protocol. Alternatively, an explanation of why a DMC is not needed	7

	21b	Description of any interim analyses and stopping guidelines, including who will have access to these interim results and make the final decision to terminate the trial	NA
Harms	22	Plans for collecting, assessing, reporting, and managing solicited and spontaneously reported adverse events and other unintended effects of trial interventions or trial conduct	7
Auditing	23	Frequency and procedures for auditing trial conduct, if any, and whether the process will be independent from investigators and the sponsor	7
**Ethics and dissemination**			
Research ethics approval	24	Plans for seeking research ethics committee/institutional review board (REC/IRB) approval	4
Protocol amendments	25	Plans for communicating important protocol modifications (eg, changes to eligibility criteria, outcomes, analyses) to relevant parties (eg, investigators, REC/IRBs, trial participants, trial registries, journals, regulators)	4
Consent or assent	26a	Who will obtain informed consent or assent from potential trial participants or authorised surrogates, and how (see Item 32)	5
	26b	Additional consent provisions for collection and use of participant data and biological specimens in ancillary studies, if applicable	5
Confidentiality	27	How personal information about potential and enrolled participants will be collected, shared, and maintained in order to protect confidentiality before, during, and after the trial	7,11
Declaration of interests	28	Financial and other competing interests for principal investigators for the overall trial and each study site	11
Access to data	29	Statement of who will have access to the final trial dataset, and disclosure of contractual agreements that limit such access for investigators	NA

For peer review only

Ancillary and post-trial care	30	Provisions, if any, for ancillary and post-trial care, and for compensation to those who suffer harm from trial participation	NA
Dissemination policy	31a	Plans for investigators and sponsor to communicate trial results to participants, healthcare professionals, the public, and other relevant groups (eg, via publication, reporting in results databases, or other data sharing arrangements), including any publication restrictions	11
	31b	Authorship eligibility guidelines and any intended use of professional writers	NA
	31c	Plans, if any, for granting public access to the full protocol, participant-level dataset, and statistical code	NA
**Appendices**			
Informed consent materials	32	Model consent form and other related documentation given to participants and authorised surrogates	Additional files
Biological specimens	33	Plans for collection, laboratory evaluation, and storage of biological specimens for genetic or molecular analysis in the current trial and for future use in ancillary studies, if applicable	NA

*It is strongly recommended that this checklist be read in conjunction with the SPIRIT 2013 Explanation & Elaboration for important clarification on the items. Amendments to the protocol should be tracked and dated. The SPIRIT checklist is copyrighted by the SPIRIT Group under the Creative Commons "Attribution-NonCommercial-NoDerivs 3.0 Unported" license.

For peer review only
